# Cancer-Associated Fibroblasts Promote Lymphatic Metastasis in Cholangiocarcinoma *via* the PDGF-BB/PDGFR-β Mediated Paracrine Signaling Network

**DOI:** 10.14336/AD.2023.0420

**Published:** 2024-02-01

**Authors:** Jian Yan, Gang Xiao, Caini Yang, Qinqin Liu, Cui Lv, Xianhuan Yu, Ziyu Zhou, Shusheng Lin, Zhenhua Bai, Haoming Lin, Rui Zhang, Chao Liu

**Affiliations:** ^1^Department of Biliary-Pancreatic Surgery, Sun Yat-sen Memorial Hospital, Sun Yat-sen University, Guangzhou, China.; ^2^Department of Thoracic Surgery, Guangzhou First People's Hospital, South China University of Technology, Guangzhou, China.; ^3^Center for Medical Research on Innovation and Translation, Guangzhou First People's Hospital, South China University of Technology, Guangzhou, China.; ^4^Guangdong Provincial Key Laboratory of Malignant Tumor Epigenetics and Gene Regulation, Guangdong-Hong Kong Joint Laboratory for RNA medicine, Sun Yat-sen Memorial Hospital, Sun Yat-sen University, Guangzhou, China.; ^5^Medical Research Center, Sun Yat-sen Memorial Hospital, Sun Yat-sen University, Guangzhou, China

**Keywords:** Cancer-associated fibroblasts (CAFs), Cholangiocarcinoma (CCA), Platelet-derived growth factor receptor beta (PDGFR-β);, lymphatic endothelial cells (LECs), lymphangiogenesis

## Abstract

Patients with cholangiocarcinoma (CCA) with lymph node metastasis (LNM) have the worst prognosis, even after complete resection; however, the underlying mechanism remains unclear. Here, we established CAF-derived PDGF-BB as a regulator of LMN in CCA. Proteomics analysis revealed upregulation of PDGF-BB in CAFs derived from patients with CCA with LMN (LN^+^CAFs). Clinically, the expression of CAF-PDGF-BB correlated with poor prognosis and increased LMN in patients with CCA, while CAF-secreted PDGF-BB enhanced lymphatic endothelial cell (LEC)-mediated lymphangiogenesis and promoted the trans-LEC migration ability of tumor cells. Co-injection of LN^+^CAFs and cancer cells increased tumor growth and LMN *in vivo*. Mechanistically, CAF-derived PDGF-BB activated its receptor PDGFR-β and its downstream ERK1/2-JNK signaling pathways in LECs to promote lymphoangiogenesis, while it also upregulated the PDGFR-β-GSK-P65-mediated tumor cell migration. Finally, targeting PDGF-BB/PDGFR-β or the GSK-P65 signaling axis prohibited CAF-mediated popliteal lymphatic metastasis (PLM) *in vivo*. Overall, our findings revealed that CAFs promote tumor growth and LMN via a paracrine network, identifying a promising therapeutic target for patients with advanced CCA.

## INTRODUCTION

Cholangiocarcinoma (CCA) is a highly lethal malignant tumor with limited treatment options [1]. It is usually asymptomatic in the early stages and is frequently not diagnosed until the disease has reached an advanced stage, resulting in poor prognosis [2]. Only one-third of patients with CCA are eligible for curative surgical excision because of a propensity for early lymph node metastasis (LNM) [3]. Several studies showed that lymphatic vessels undergo dynamic changes within the tumor, including tumor-associated lymphangiogenesis, which facilitate metastasis [4], while the formation of intratumoral lymphatic vessels has been shown to play an important role in tumor metastatic dissemination [5]. However, the underlying mechanism of lymphatic metastasis in CCA remains poorly understood.

CCA is a highly desmoplastic neoplasm whose tumor microenvironment (TME) contains many myofibroblasts (MFBs) or cancer-associated fibroblasts (CAFs), which contribute to its progression, as has been proposed for other tumors (e.g., prostate cancer and breast cancer) [6-7] In CCA, the abundance of CAFs correlates positively with tumor growth and poor survival [8]. CAFs secrete a variety of signaling molecules (such as interleukin 1 beta (IL-1β), platelet-derived growth factor beta homodimer (PDGF-BB) and stromal cell-derived Factor 1 (SDF1)), which promote tumor progression by enhancing proliferation, migration, invasion, chemotaxis, and intratumoral angiogenesis [9]. Furthermore, some studies have shown that CAFs can promote CCA growth through short-range and direct cell-cell morphogenetic signals such as hedgehog and notch receptor 3 (NOTCH3) [10-11]. CAFs can also secrete certain immunomodulatory factors that contribute to the immunosuppressive TME, for example, they regulate innate immunity by reducing natural killer (NK) cell activation and supporting M2 macrophages [12]. Massimiliano *et al.* found that CCA-derived PDGF stimulates CAFs to secrete certain growth factors and targeting CAFs or PDGF-D-induced signals might be an effective tool to reduce the invasiveness of CCA and block cancer-associated lymphangiogenesis [13]. Most recent research investigating cancer-associated lymphangiogenesis has focused on vascular endothelial growth factor (VEGF) family members, which can interact with the receptor VEGFR-3 on lymphatic endothelial cells [14]. In addition to VEGF family members, Coltrera *et al.* found that tumors with high lymphatic metastatic ability also express other growth factors, such as PDGF-BB in breast cancer tissues [15]. The PDGF family includes five structurally related members, PDGF-AA, -BB, -AB, -CC, and -DD, which are present as both homodimers and heterodimers. PDGF-BB is the only ligand that can activate both the platelet-derived growth factor receptor (PDGFR)-α and PDGFR-β. Renhai *et al.* found that PDGF-BB induces intratumoral lymphangiogenesis and promotes lymphatic metastasis in fibrosarcoma [16]. Interestingly, a recent study showed that CAF-derived PDGF-BB might modulate Hedgehog survival signaling in CCA cells [17]; however, it has not yet been explored whether CAF-derived PDGF-BB directly induces intratumoral lymphangiogenesis and promotes LNM in CCA.

The objective of this study was to evaluate whether CAF-derived PDGF-BB plays a direct role in promoting lymphangiogenesis and LNM of CCA. The results suggested that PDGF-BB secreted by CAFs can promote the proliferation, migration, and invasion of PDGFR-β-positive CCA cell lines, and the lymphangiogenic formation potential of lymphatic endothelial cells (LECs). Our data demonstrated that PDGF-BB secreted by CAFs induces tumor lymphangiogenesis and LNM and suggested that growth factors other than VEGFs play a key role in these processes. Thus, targeting PDGFR-β in conjunction with inhibitors of downstream pathways will provide a more effective means to block tumor growth and lymphangiogenic metastasis of CCA.

## MATERIALS AND METHODS

### Patients and clinical samples

A total of 126 tumor tissue samples from patients with CCA who underwent radical surgery at The Second Affiliated Hospital of Sun Yat-sen University between January 2012 and July 2018 were obtained for this study. Blood samples were obtained from another 20 patients with CCA and 20 participants without CCA. Patients in the CCA group were eligible for inclusion if they had pathologically confirmed CCA. The clinical features of the patients with CCA are summarized in Supplementary Table 1. All the experiments were approved by the Sun Yat-sen University Human Research Ethics Review Committee.

### Immunohistochemistry (IHC)

Antigens were extracted from 4-mm thick paraffin-embedded sections in 0.01 M trimethylol aminomethane salt liquid acid (pH = 9.2) for 15-20 min in a pressure cooker to remove the aldehyde junctions formed during the initial tissue fixation. The samples were incubated at 4 °C overnight with specific antibodies against alpha smooth muscle actin (α-SMA) (Servicebio, GB13044), PDGF-BB (ThermoFisher, PA588272), podoplanin (Novus, NBP1-90211), lymphatic vessel endothelial hyaluronan receptor 1 (LYVE1) (Abcam, Ab281587), PDGFR-β (Signalway Antibody, 41327), PDGFR-α (Signalway Antibody, 33470), phosphorylated (p)-focal adhesion kinase (p-FAK) (Signalway Antibody, C90918Bio), FAK (Cell Signaling Technology, 3285), p-protein kinase B (p-AKT) (Cell Signaling Technology, 13038), AKT (Cell Signaling Technology, 2920), p-P65 (Abcam, Ab86299), P65 (Cell Signaling Technology, 8242), p-JUN N-terminal kinase (p-JNK) (Cell Signaling Technology, 4668), JNK (Abcam, Ab76125), p-extracellular regulated kinase (p-ERK) (Cell Signaling Technology, 4370), ERK (Cell Signaling Technology, 4696), p-glycogen synthase kinase (p-GSK) (Cell Signaling Technology, 9336), GSK (Cell Signaling Technology, 12456), p-yes associated protein (p-YAP) (Cell Signaling Technology, 13008), YAP (Cell Signaling Technology, 14074), or marker of proliferation Ki-67 (Ki-67) (Servicebio, GB111499) and stained the following day using diaminobenzidine (Dako) according to the manufacturer’s instructions. Details of all antibodies are given in Supplementary Table 2.

### Immunofluorescence (IF)

For tissue IF, the antigens were extracted as described in the IHC assay. Tissue sections were blocked with Tris-buffered saline-Tween 20 (TBST) containing 5% bovine serum albumin (BSA) (Sigma-Aldrich, V900933) for 1 h at room temperature (RT). Subsequently, the tissue sections were blocked in TBST containing 5% BSA for 1 h at RT. After blocking, the samples were incubated overnight at 4 °C with primary antibodies followed by incubation with Alexa Fluor-conjugated secondary antibodies (Invitrogen) for 1 h at RT. Nuclei were counterstained using 4′,6-diamidino-2-phenylindole (DAPI, Servicebio, G1012). For cellular IF, cells were seeded into confocal cell culture dishes and fixed using 4 % formaldehyde, permeabilized with 0.2% Triton X-100, and blocked with 5% BSA for 1 h at RT. Subsequently, the samples were incubated with primary antibodies at 4 °C overnight and then with secondary antibodies at RT for 1h. Finally, the nuclei were stained with DAPI. Images were captured using a laser confocal microscope (LSM780, Zeiss). Details of all antibodies are given in Supplementary Table 2.

### Multiplex tyramide signal amplification (TSA) staining

For double fluorescence staining of primary antibodies of the same species, TSA staining was performed. First, the antigens were extracted as described in the IHC assay. The tissue sections were treated with 3% H_2_O_2_ for 20 min to eliminate endogenous peroxidase activity, and permeated with 0.2% Triton X-100, and blocked with 5% BSA for 1 h at RT. After blocking, the samples were incubated overnight at 4 °C with primary antibodies followed by incubation with corresponding second antibody. After thorough cleaning with TBST, signal amplification was achieved using a specific TSA-AF488 (Invitrogen, B40953) or TSA-AF555 (Invitrogen, B40955) dye and terminated with PBS containing 100 mM benzoyl hydrazine and 50 mM H_2_O_2_. Tissue sections were then immersed in eluent to remove unbound antibodies and then re-blocked. Next, the sections were stained with additional primary antibodies. The next step is the same as before. After all antibodies were stained, DAPI staining was finally used. The cross sections were imaged under fluorescence microscopy.

### Popliteal lymph node metastasis (PLM) model

BALB/c nude mice (4 weeks old, 18-20 g) were purchased from the Guangdong Province Experimental Animal Center. All experimental procedures were approved by the Sun Yat-sen University Institutional Animal Care and Use Committee. 5 × 10^6^ tumor cells (QBC939^Luci^ or HuCCT^Luci^), alone or with 5 × 10^5^ CAFs, such as LN^+^ CAFs, LN^-^ CAFs, and *PDGFB*-knockout CAFs (CAFs*^PDGFB^*^(KO)^), were resuspended in 50 μl of PBS and inoculated into the foot pad from the toe junction of the hind limb. For the treatment assays, anti-PDGF-BB antibodies (50 μg/kg in PBS), isotype control IgG (50 μg/kg in PBS), a PDGFR inhibitor (STI571) (50 mg/kg), and blank control dimethyl sulfoxide (DMSO) (2%) were injected intraperitoneally (IP) every 3 days. *In vivo* imaging was performed six weeks after injection using a bioluminescence imaging system (IVIS Spectrum Imaging System, PerkinElmer). The primary tumor and popliteal lymph nodes (PLNs) were removed, paraffin-embedded, and 4 μm serial sections were obtained for hematoxylin-eosin (H&E) staining and IHC analysis. Images were captured using an Eclipse 80I system and the NIS-Elements software (Nikon).

### Subcutaneous tumor models

Cells used for injection were resuspended in an equal volume of PBS and Matrigel (BD Biosciences) solution. 2 × 10^6^ CCA cells (QBC939 or HuCCT1) were inoculated into nude mice by IP to detect tumor growth. In the co-injection groups, the nude mice were subcutaneously injected with both 2 × 10^6^ CCA cells and either 2 × 10^5^ LN^+^ CAFs, LN^-^ CAFs, or CAFs*^PDGFB^*^(KO)^. Tumor growth was recorded weekly using calipers. The tumor volume was calculated as (length × width^2^)/2. When the mean tumor volume reached 100 mm^3^, the mice were treated with PDGF-BB antibodies, the P65 inhibitor (P65i) PG490 (Applied InvivoChem Inc.; 1 mg/kg, IP), the GSK inhibitor (GSKi) CHIR-98014 (Applied AOBIOUS Inc.; 20 mg/kg, IP), or STI571 (Applied Selleck Inc.) (50 mg/kg, IP), with DMSO and isotype IgG were used as treatment controls, every 3 days for up to 2-3 weeks. At the end point of the trial, all mice were euthanized, and the tumors were removed, fixed overnight in 4% formaldehyde in PBS, and then analyzed by H&E staining and immunostaining.

### Enzyme-linked immunosorbent assay (ELISA)

Primary CAFs were cultured in Dulbecco’s modified Eagle’s medium (DMEM) containing 10% fetal bovine serum (FBS) until 80% confluent. The cells were washed 2-3 times with PBS and cultured in fresh serum-free medium with 5% CO_2_ at 37 °C for 24 h. The cell culture supernatant was collected 24 h later for ELISA. PDGF-BB ELISA kits were purchased from Enzyme linked Biotechnology Co. (MIbio, ml001574J, Shanghai, China). All experimental procedures were carried out in accordance with the product instructions.

**Figure 1.**
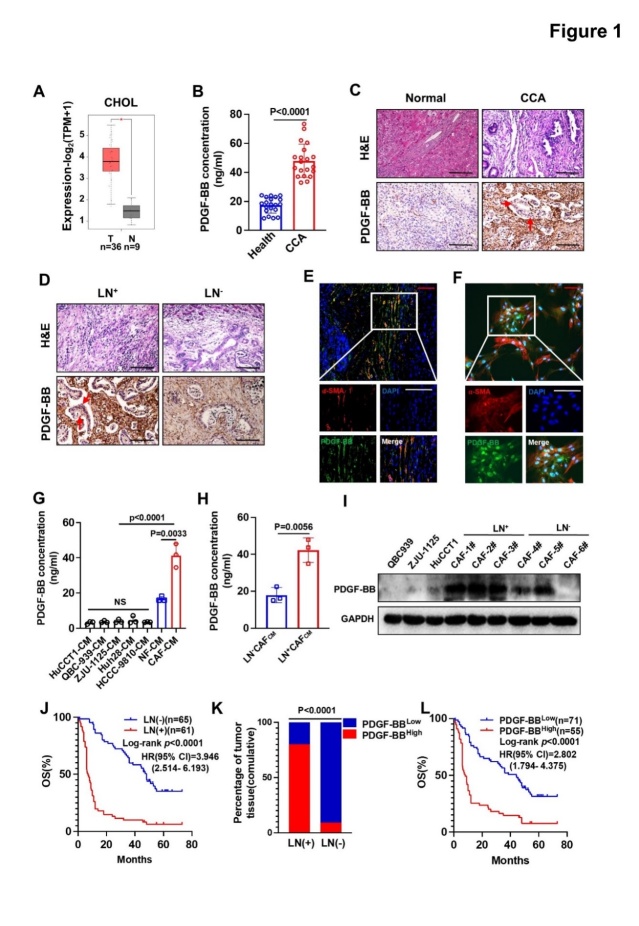
**CAF-derived PDGF-BB correlated with LNM in CCA**. (**A**) GEPIA database analysis of the relative *PDGFB* mRNA expression in matched CCA tissues (n = 36) and adjacent non-CCA tissues (n = 9). (**B**) Secreted PDGF-BB concentration in the serum of 20 patients with CCA and 20 non-cancer controls (Healthy). (**C**) Representative images of PDGF-BB immunohistochemical staining in human CCA and non-cancer tissues. The red arrows represent cancer cells. (**D**) Representative images of PDGF-BB immunohistochemical staining in LN^+^ and LN^-^ CCA tissues. (**E**) Representative images of PDGF-BB and α-SMA double-labeled immunofluorescent staining in CCA tissues. (**F**) Representative images of PDGF-BB and α-SMA double-labeled immunofluorescent staining in CAFs. (**G**) Secreted PDGF-BB concentration in CCA cell lines and myofibroblast supernatants. (**H**) Secreted PDGF-BB concentration in LN^+^ and LN^-^ CAF supernatants. (**I**) Western blotting results of PDGF-BB levels in LN^+^ and LN^-^ CAFs. (**J**) OS of patients with LN^+^ CCA (n = 61) and LN^-^ CCA (n = 65). (**K**) The percentages of CCA tissue specimens from 126 patients with high and low PDGF-BB levels. (**L**) OS of patients with PDGF-BB-high (n = 55) and PDGF-BB-low (n = 71) expression. All *in vitro* experiments were performed with three biological replicates or three independent experiments. The error bars indicate the means ± SD of three independent experiments. Statistical method: (B) The nonparametric Mann-Whitney *U* test, (H) Student’s t test, (G) One-way ANOVA followed by Dunnett’s tests, (K) χ^2^ test. (J, L) Kaplan-Meier method. Scale bars: (C, D) represent 200 μm, (E, F) red, 50 μm, white 100 μm. Abbreviations: GEPIA, Gene Expression Profiling Interactive Analysis; CAFs, cancer-associated fibroblasts; PDGF-BB, platelet derived growth factor subunit B homodimer; LNM, lymph node metastasis; CCA, cholangiocarcinoma; LN, lymph node; α-SMA, alpha smooth muscle actin; OS, overall survival.

### Proteomic analysis

For sample preparation, LN^+^CAFs and LN^-^CAFs were cultured in 100 mm culture dishes with DMEM in a 5% CO_2_ cell incubator at 37 °C. When the cells reached 80% confluence, the medium was changed to serum-free DMEM and after 24 h of culture, the cell culture supernatant was harvested, and the total protein content was determined using a Pierce™ BCA Protein Assay Kit. Then, the supernatants were precipitated with acetone, resuspended in 50 mM urea buffer, reduced with dithiothreitol (DTT, 2 mM, 37 °C for 1.5 h), alkylated with iodoacetamide (IAM, 10 mM, 25 °C 40 min), and diluted to 60 mM urea for overnight digestion with trypsin (Sequencing Grade Modified Trypsin, V5111, Promega, Madison, WI, USA) at 37 °C. The samples were then diluted two-fold again and digested overnight with trypsin at 37 °C. The tryptic peptides were desalted using an HLB column (Oasis HLB, 186000383, Waters, Milford, MA, USA) and evaporated to dryness. In all, 300 μg of dried protein from LN^+^CAFs and LN^-^CAFs digests were dissolved in 10 μl of 0.1% formic acid with H_2_O). The subsequent mass spectrometer operation procedures are described in a previous study [18].

### Statistics

A t-test and one-way analysis of variance (ANOVA) were used for normally distributed data. The Mann-Whitney U test was used for non-normally distributed data. Quantitative data are presented as the mean ± SD of at least three independent experiments. The threshold of significance was set at p < 0.05. The patient samples were divided into two groups based on their mean expression levels before survival analysis. The relationship between protein expression and prognosis was evaluated using Kaplan-Meier survival analysis. P-values were calculated using the log-rank test.

## RESULTS

### Expression levels of PDGF-BB and PDGFR-β were upregulated in CCA and were associated with LNM

To determine the clinical importance of PDGF-BB expression in CCA, we first analyzed the TCGA-CHOL dataset by using the Gene Expression Profiling Interactive Analysis (GEPIA) website. The results indicated that *PDGFB* has higher expression in CCA tumors than in normal controls (Fig. 1A). Moreover, ELISA tests verified that patients with CCA had higher serum PDGF-BB levels than the healthy controls (Fig. 1B). In addition, IHC staining showed that PDGF-BB was mainly expressed in fibroblast-like stromal cells, and PDGF-BB levels were higher in the tumors of the patients with node-positive CCA (LN^+^CCA) than in those with node-negative CCA (LN^-^CCA) (Fig. 1C-D). Next, multiple fluorescence staining was performed to identify the primary cells producing PDGF-BB. The results showed that α-SMA and PDGF-BB co-localized significantly, indicating that CAFs were the main source of PDGF-BB in CCA (Fig. 1E-F).

To further confirm the fibroblast origin of PDGF-BB, we isolated primary fibroblasts from CCA tissue and adjacent normal tissue. Flow cytometry analysis (Supplementary Fig. 1A), immunostaining (Supplementary Fig. 1B), and western blotting (Supplementary Fig. 1C) experiments showed that the fibroblasts expressed α-SMA, FAP, but did not express endothelial cell marker CD31, immune cell marker CD45, or epithelial cell marker CK19, demonstrating that the fibroblasts were highly purified, and thus could be used as cancer-associated fibroblast (CAF) and normal fibroblast (NF) cell lines in subsequent experiments. Furthermore, ELISA (Fig. 1G-H) and western blotting (Fig. 1I) experiments additionally confirmed that PDGF-BB was mainly expressed in primary CAFs rather than normal fibroblasts (NFs) and CCA cell lines, including QBC939, HuCCT1, Huh28, HCCC-9810, and ZJU-1125. In addition, we compared the cytokine secretion profiles of LN^+^CAFs and LN^-^CAFs using mass spectrometry (MS) and found that PDGF-BB was one of the most significantly upregulated cytokines in LN^+^CAFs (Supplementary Fig. 2A-B). Notably, the level of PDGF-BB was higher in CAFs isolated from patients with node-positive CCA (LN^+^CAFs) than from patients with node-negative CCA (LN^-^CAFs) (Fig. 1H-I), which was consistent with the MS and IHC results. Consistent with a previous report [19], our data also suggest that LNMs was a poor prognostic factor for CCA (Fig. 1J). Considering the elevated expression of PDGF-BB in LN^+^CAFs, we analyzed the correlation between PDGF-BB and node status (Fig. 1K). The results showed that LN^+^CCA had a higher level of PDGF-BB, and elevated PDGF-BB correlated with patient outcome in the Kaplan-Meier survival analysis (Fig. 1L). These results suggested that CAF-derived PDGF-BB might be involved in CCA LNM.

**Figure 2.**
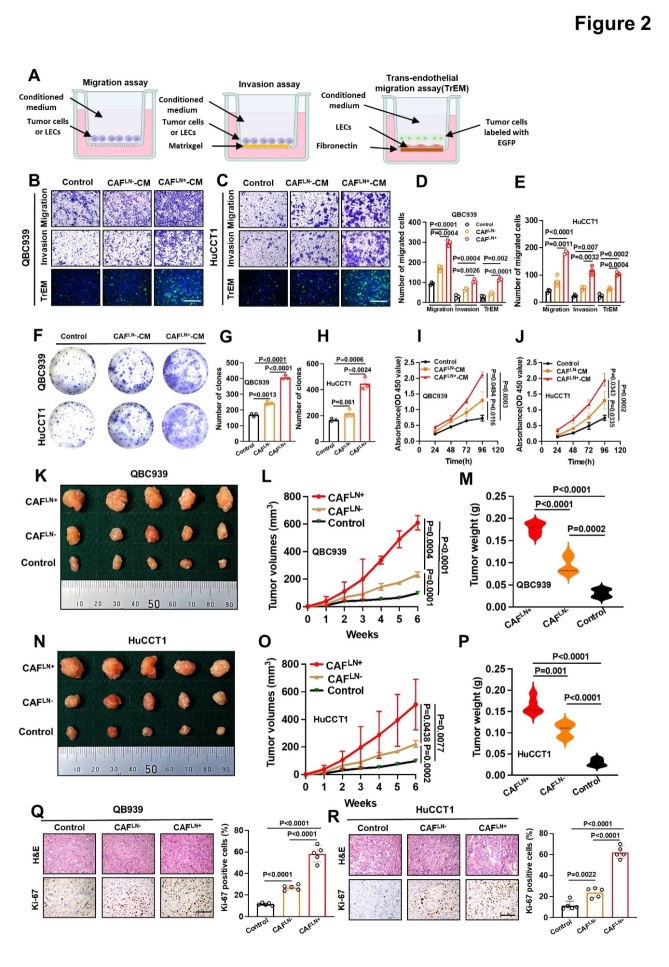
**LN^+^ CAFs strongly promoted CCA cell aggressiveness *in vitro* and *in vivo***. (**A**) Schematic representation of the establishment of the Transwell assay. (B, C) Representative images of the migration, invasion, and TrEM of QBC939 (B) and HuCCT1 cells (C) treated with LN^+^ or LN^-^ CAF supernatants (magnification: ×100). (D, E) Histogram analysis of the migration, invasion, and TrEM of QBC939 (D) and HuCCT1 cells (E). (F-H) Representative images (F) and histogram analysis of colony formation by QBC939 (G) and HuCCT1 (H) cells. (I, J) MTT assays to measure the proliferation of QBC939 (I) and HuCCT1 cells (J) incubated with LN^+^ or LN^-^ CAF supernatants. (K, N) Representative images of QBC939 (K) and HuCCT1 (N) cells combined with LN^+^ or LN^-^ CAFs on nude mouse xenografts. (L, M) QBC939 tumor volumes (K) and weights (L) (n = 5). (N, O) HuCCT1 tumor volumes (O) and weights (P) (n = 5). (Q, R) Representative images and histogram analysis of immunohistochemical staining for Ki67 expression in QBC939 (Q) and HuCCT1 (R) cells (n = 5). All *in vitro* experiments were performed with three biological replicates or three independent experiments. The error bars indicate the means ± SD of three independent experiments. Statistical method: (D-E, G-H, M, P, Q-R) One-way ANOVA, (I-J, L, O) Two-way ANOVA. Scale bars: (B, C)100 μm, (Q, R) 200 μm.

We then evaluated the expression of classical PDGF-BB receptors in CCA. GEPIA analysis of the TCGA-CHOL dataset showed no difference in *PDGFRA* expression between normal and tumor tissues, while *PDGFRB* was significantly upregulated in tumors, suggesting that PDGFR-β is the major functional receptor of PDGF-BB in CCA (Supplementary Fig. 3A-B). We noticed that PDGFR-β expression was significantly higher in LN^+^CCA than in LN^-^CCA and the normal controls (Supplementary Fig. 3C). Meanwhile, there was no obvious staining signal of PDGFR-α in normal or CCA tissues, regardless of lymph node metastasis (LNM) (Supplementary Fig. 3D), and the detection of PDGFR-α and PDGFR-β in cell lines was consistent with this observation (Supplementary Fig. 3G-H). Dual immunofluorescence showed that PDGFR-β was expressed in both tumor cells (CK19^+^) and LECs (labeled by PDPN, LYVE-1 or PROX-1) (Supplementary Fig. 3E-F). PDPN, LYVE-1, and PROX-1 are recognized markers of lymphatic endothelial cells [13, 20]. IHC and IF experiments were performed, and the results showed that the expression distribution of PROX-1, PDPN, and LYVE-1 basically overlapped, indicating that PDPN and LYVE-1 are suitable as lymphatic markers for CCA Supplementary (Fig. 4A-C). Moreover, according to the intensity and area of the PDGFR-β staining signal (Supplementary Fig. 5A-B), our cohort was divided into Epithelium^High^ and Epithelium^Low^, or Stroma^Negative/Low^ and Stroma^Median/High^ groups. Among them, the Epithelium^High^ patients accounted for 66.67% (84/126), and had the worst overall survival (OS) (Supplementary Fig. 5C). Notably, patients with high PDGFR-β expression in both tumor cells and stromal cells had the worst prognosis, while patients with low PDGFR-β expression in both types of cells had the best survival (Supplementary Fig. 5D), indicating the presence of a synergistic effect of PDGFR-β expression on tumor cells and stromal cells for patient survival. Furthermore, chi-squared tests showed a correlation between lymph node status and PDGFR-β expression in both epithelial and stromal cells, while tumor diameter only correlated with PDGFR-β expression in epithelial cells, but not in stroma cells (Supplementary Fig. 5E-H). Moreover, GEPIA analysis of TCGA data showed that *PDGFB* (Supplementary Fig. 6A) and *PDGFRB* (Supplementary Fig. 6B) were significantly overexpressed in various types of human cancers. The database showed that both *PDGFB* and *PDGFRB* were highly expressed in Cholangiocarcinoma (CHOL), Liver hepatocellular carcinoma (LIHC), Lymphoid Neoplasm Diffuse Large B-cell Lymphoma (DLBC), Pancreatic adenocarcinoma (PAAD and Head and Neck squamous cell carcinoma (HNSC) (Supplementary Fig. 6C-D). Multivariate Cox regression analysis was performed to adjust for established clinical risk factors, such as nerve infiltration, grade, stage, hepatic capsule invasion, and lymph node status, among others, suggesting that stromal expression of PDGFR-β could be an independent prognostic factor for poor survival in CCA (Supplementary Table 3).

### LN^+^CAFs strongly promote the aggressiveness of CCA cells in vitro and in vivo

LNM is a complex multi-step process. In addition to cancer-associated lymphangiogenesis *in vivo*, enhanced cell migration and invasion are essential for LNM [21-22]. To uncover the role of CAFs in CCA progression *in vitro*, transwell (Fig. 2A), colony formation, and cell counting kit 8 (CCK8) assays were performed to examine the effects of LN^+^CAF and LN^-^CAF supernatants on the migration, invasion, trans-endothelial migration (TrEM), clonal formation, and proliferation of QBC939 and HuCCT1 CCA cells. The results showed that LN^+^CAFs had the strongest ability to promote migration, invasion, TrEM, clone formation, and proliferation in both CCA cell lines compared with those of the LN^-^CAF supernatant and medium control (Fig. 2B-J). Consistent with the *in vitro* findings, both cancer cells injected alone, and cancer cells co-injected with LN^-^CAFs showed slower growth in terms of tumor size and weight compared with those of the LN^+^CAF co-injection group (Fig. 2K-P). The changes in tumor volume and weight might be attributed to differences in the level of cell proliferation, because the LN^+^CAF co-injection group had the highest proportion of Ki67-positive tumor cells (Fig. 2Q-R). These *in vivo* and *in vitro* results suggested that LN^+^CAFs contribute to CCA progression by promoting several aspects, such as proliferation and migration.

**Figure 3.**
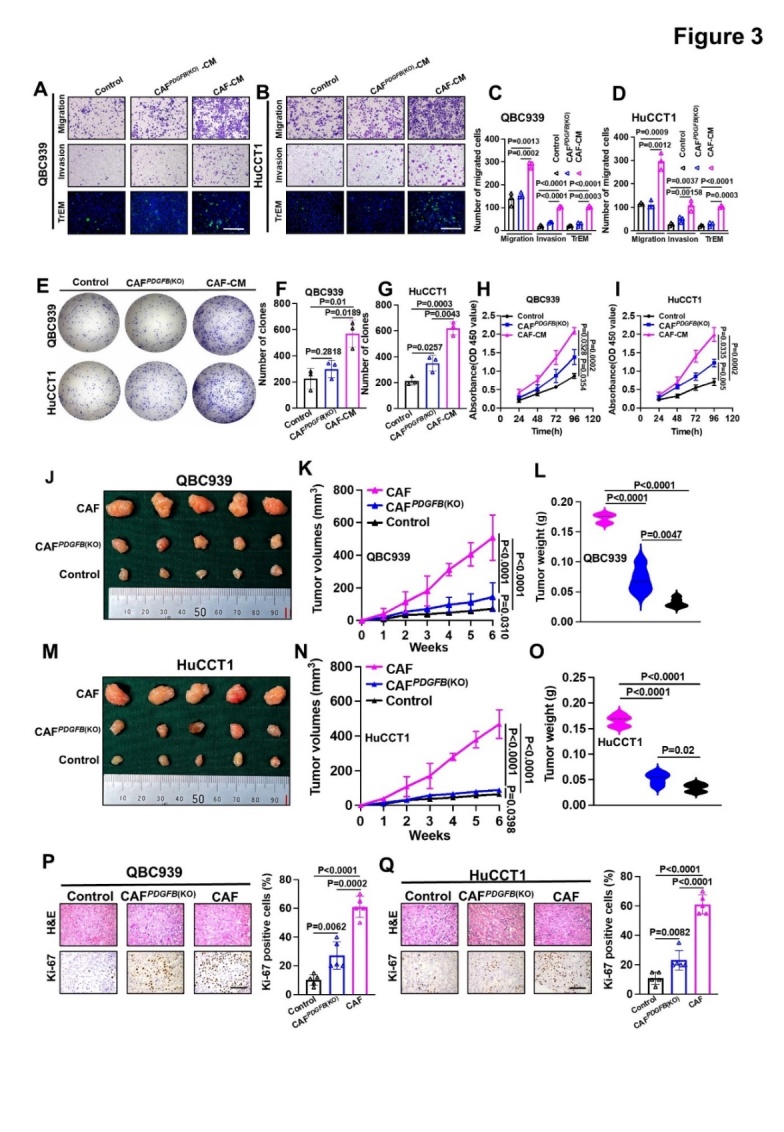
***PDGFB* knockout in CAFs reduced the promoting effect on CCA cells *in vitro* and *in vivo***. (A, B) Representative images of migration, invasion, and TrEM assays of QBC939 (A) and HuCCT1 cells (B) treated with supernatant from CAFs or CAFs*^PDGFB^*^(KO)^. (C, D) Histogram analysis of migration, invasion, and TrEM of QBC939 (C) and HuCCT1 cells (D). (E-G) Representative images (E) and histogram analysis of colony formation by QBC939 (F) and HuCCT1 cells (G). (H, I) MTT assays to measure the proliferation of QBC939 (H) and HuCCT1 cells (I) incubated with CAF or CAF*^PDGFB^*^(KO)^ supernatants. (J, M) Representative images of QBC939 (J) and HuCCT1 (M) cells combined with CAFs or CAF*^PDGFB^*^(KO)^ on nude mouse xenografts. (K, L) QBC939 tumor volumes (K) and weights (L) (n = 5). (N, O) HuCCT1 tumor volumes (N) and weights (O) (n = 5). (P, Q) Representative images and histogram analysis of immunohistochemical staining of Ki67 expression in QBC939 (P) and HuCCT1 cells (Q) (n = 5). All *in vitro* experiments were performed with three biological replicates or three independent experiments. The error bars indicate the means ± SD of three independent experiments. Statistical method: (C-D, F-G, L, O, P-Q) One-way ANOVA, (F-G, K, N) Two-way ANOVA. Scale bars: (A, B)100 μm, (P, Q) 200 μm.

**Figure 4.**
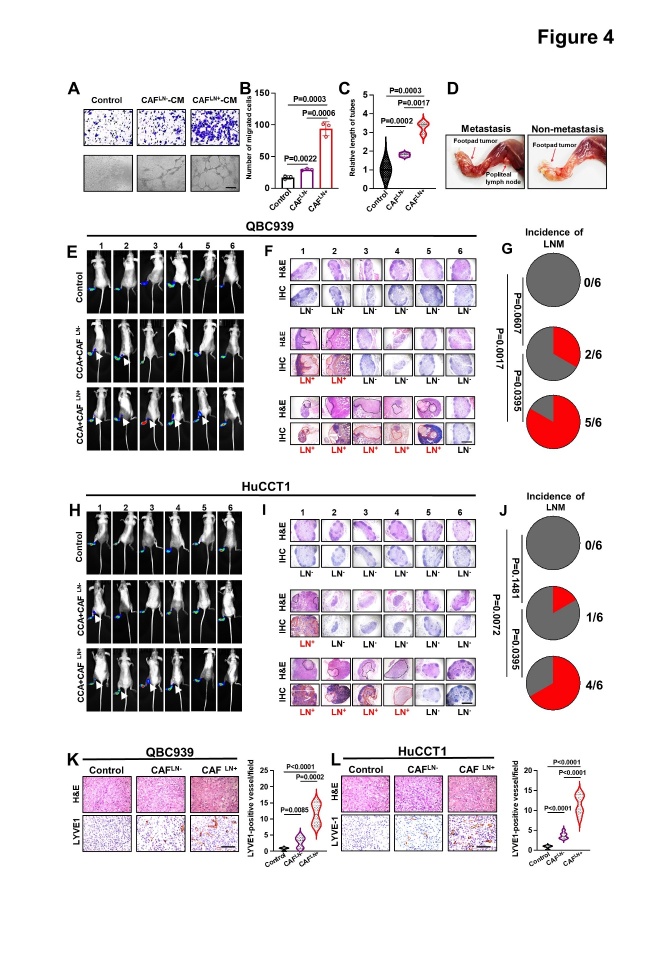
**LN^+^ CAFs promoted marked lymphangiogenesis and LNM in CCA**. (A-C) Representative images (A) and quantification of Transwell migration (B) and tube formation (C) by HLECs treated with supernatants from LN^+^ or LN^-^ CAFs. (**D**) Representative gross anatomy images of the PLNM and non-LNM nude mouse model with QBC939 cells. (E, H) Representative images of bioluminescence analysis (PLNs) of footpad tumors and PLNM in QBC939 (E) and HuCCT1 (H) cells (n = 6 per group). (F, I) Enucleated PLNs and immunohistochemical staining with anti-luciferase antibodies in QBC939 (F) and HuCCT1 (I) cells (n = 6 per group). Scale bars: red, 50 μm. (G, J) Pie chart analysis of the PLNM rate of QBC939 (G) and HuCCT1 cells (J). (K-L) Representative images of intratumoral microlymphatic vessels stained with anti-LYVE1 (left) and histogram quantification of LMVD (right) in the QBC939 (K) and HuCCT1 (L) groups. All *in vitro* experiments were performed with three biological replicates or three independent experiments. The error bars indicate the means ± SD of three independent experiments. Statistical method: (B-C, K-L) One-way ANOVA, (G, J) χ^2^ test. Scale bars: (A) 100 μm, (F, I) 50 μm, (K, L) 200 μm.

**Figure 5.**
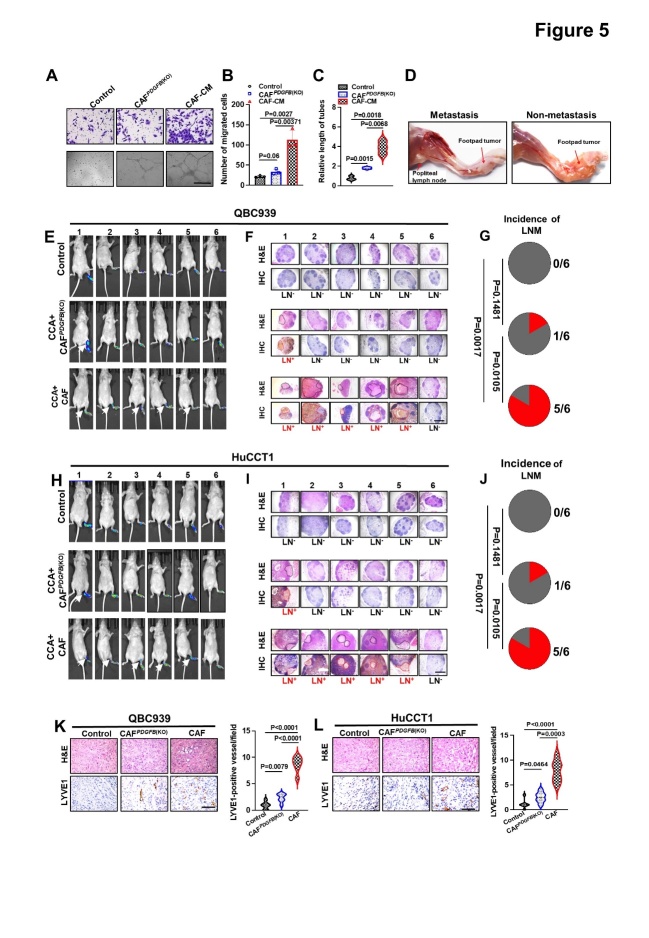
***PDGFB* knockout in CAFs reduced the promoting effect on lymphangiogenesis and LNM in CCA**. (A-C) Representative images (A) and quantification of Transwell migration (B) and tube formation (C) by HLECs treated with CAF and CAF*^PDGFB^*^(KO)^ supernatants. (**D**) Representative gross anatomy images of the PLNM and non-LNM nude mouse model. (E, H) Representative images of the bioluminescence analysis of footpad tumors and PLNM in the QBC939 (E) and HuCCT1 groups (H) (n = 6 per group). (F, I) Enucleated LNs and immunohistochemical staining with anti-luciferase antibodies in QBC939 (F) and HuCCT1 (I) (n = 6 per group). (G, J) Pie chart analysis of PLNM rate of QBC939 (G) and HuCCT1 cells (J). (K-L) Representative images of intratumoral microlymphatic vessels stained with anti-LYVE1 (left) and histogram quantification of LMVD (right) in the QBC939 (K) and HuCCT1 (L) groups. All *in vitro* experiments were performed with three biological replicates or three independent experiments. The error bars indicate the means ± SD of three independent experiments. Statistical method: (B-C, K-L) One-way ANOVA, (G, J) χ^2^ test. Scale bars: (A) 100 μm, (F, I) 50 μm, (K, L) 200 μm.

**Figure 6.**
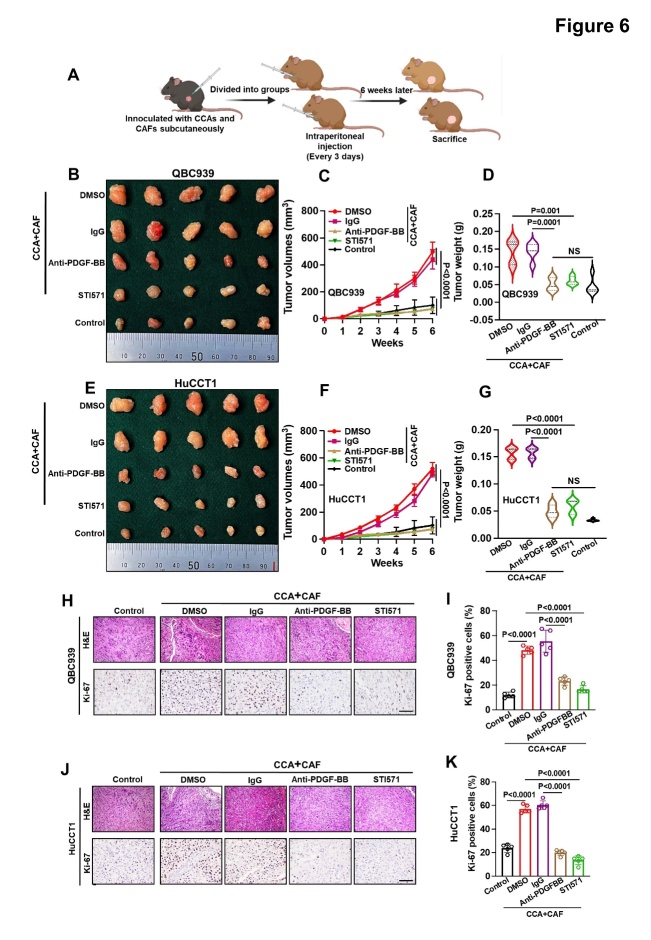
**CAF-derived PDGF-BB promoted CCA aggressiveness through PDGFR-β *in vivo***. (**A**) Schematic representation of the establishment of the xenograft model. (B, E) Representative images of QBC939 (B) and HuCCT1 cells (E) combined with CAFs on nude mouse xenografts treated with DMSO (2%), IgG (50 μg/kg), anti-PDGF-BB antibodies (50 μg/kg), or STI571 (50 mg/kg) (n = 5). (C, D) QBC939 tumor volumes (C) and weights (D) (n = 5). (F, G) HuCCT1 tumor volumes (F) and weights (G) (n = 5). (H-K) Representative images (K, J) and histogram analysis (I, K) of immunohistochemical staining for Ki67 expression (n = 5). Results are shown as means ± SEM. Statistical method: (C, F) Two-way ANOVA, (D, G, I, K) One-way ANOVA. Scale bars: 200 μm.

To uncover whether the functional role of CAFs relies on PDGF-BB secretion, we first examined the effect of recombinant human PDGF-BB on the proliferation, clonal formation, proliferation, and migration of CCA cell lines with different PDGFR-β expression levels Supplementary (Fig. 3H). As expected, PDGF-BB dramatically improved the clonal formation, proliferation, and migration abilities of PDGFR-β-positive (HuCCT1 and QBC939), but not PDGFR-β-negative, CCA cell lines (Huh28, HCCC-9810, and ZJU-1125) in a concentration-dependent manner (Supplementary Fig. 7A-E). These findings revealed that CAF-derived PDGF-BB might play a role in promoting tumor progression in CCA through the PDGF-BB/PDGFR-β signaling pathway.

**Figure 7.**
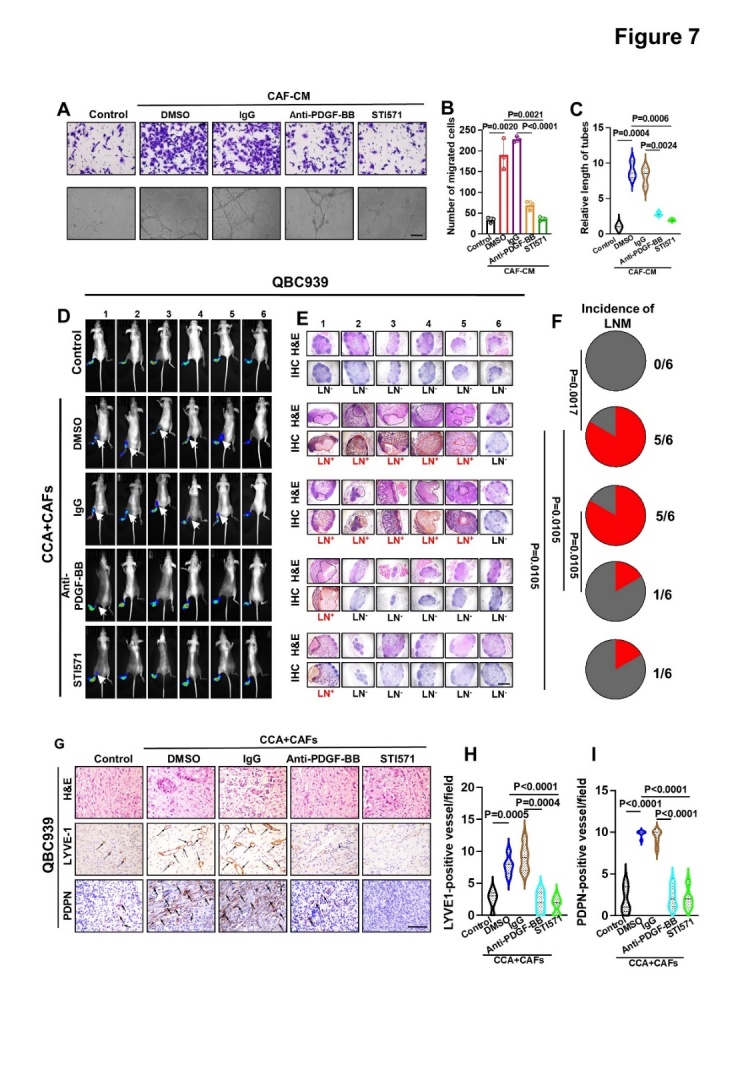
**CAFs promote lymphangiogenesis and LNM through the PDGF-BB/PDGFR-β pathway both *in vitro* and *in vivo***. (A-C) Representative images (A) and quantification of Transwell migration (B) and tube formation (C) by HLECs incubated with CAF supernatant (CAF-CM) treated with DMSO (0.1%), IgG (50 ng/ml), anti-PDGF-BB (50 ng/ml), or STI571 (100 μg/ml). (**D**) Representative images of bioluminescence analysis of footpad tumors and PLNM in QBC939 cells combined with CAFs on nude mice xenografts treated with DMSO (2%), IgG (50 μg/kg), Anti-PDGF-BB (50 μg/kg), or STI571 (50 mg/kg) (n = 6). (**E**) Enucleated PLNs and immunohistochemical staining of each PLN with anti-luciferase antibodies in the metastatic and non-metastatic groups. (**F**) Pie chart analysis of the PLNM rate of QBC939 cells. (G-I) Representative images of intratumoral microlymphatic vessels stained with anti-LYVE1 and PDPN (left) (G) and histogram quantification of LMVD (right) in the QBC939 group (H, I). All in vitro experiments were performed with three biological replicates or three independent experiments. The error bars indicate the means ± SD of three independent experiments. Statistical method: (B-C, H-I) One-way ANOVA, (F) χ^2^ test. Scale bars: (A) 100 μm, (E) 50 μm, (G) 200 μm.

To determine the necessity of PDGF-BB for CAF function, we used CRISPR/CAS9 to knockout *PDGFB* in LN^+^ CAFs, which was validated using ELISA and western blotting analysis (Supplementary Fig. 8A-C). *PDGFB* knockout markedly rescued the enhanced migration, invasion, TrEM, clonal formation, and proliferation of HuCCT1 and QBC939 cells induced by LN^+^CAFs (Fig. 3A-I). Consistent with the *in vitro* results, *PDGFB* knockout in CAFs could dramatically reduce their promotion of tumor growth *in vivo*. Compared with the CAF group, the CAF*^PDGFB^*^(KO)^ group and the control group had smaller tumor volumes and weights (Fig. 3J-O), and lower Ki67 expression levels (Fig. 3P-Q). These results suggested that PDGF-BB derived from CAFs promotes the aggressiveness of CCA cells both *in vitro* and *in vivo*.

### LN^+^CAFs considerably promote lymphangiogenesis and lymphatic metastasis in cholangiocarcinoma

Next, the distribution of CAFs and the extent of tumor-associated lymphangiogenesis were evaluated in human CCA samples. CAFs (α-SMA^+^) and lymphatic vessels (PDPN^+^ or LYVE1^+^) were abundant in CCA tumor tissues compared with those in normal liver tissues and para-tumoral tissues (Supplementary Fig. 9A, 9C), and were spatially close to each other (Supplementary Fig. 9B), suggesting an interaction between them. The intratumoral micro-lymphatic density (IMLD) is a crucial factor affecting the lymph node metastasis of tumors. We found that the IMLD was much higher in LN^+^ CCA than in LN^-^CCA Supplementary (Fig. 10A-B, 11A-B). These findings suggested that lymphangiogenesis might be associated with LNM status in CCA.

Given the agreement between the number and spatial distribution of CAFs and LECs in CCA tissues, we speculated that CAFs have the potential to induce lymphangiogenesis. LEC tube formation assays showed that the LN^+^CAF-derived supernatant induced more and larger lymphatic vessels than the LN^-^CAF supernatant and medium control (Fig. 4A, 4C). In addition, Transwell assays showed that the LN^+^CAF-derived supernatant had the strongest ability to promote LEC migration (Fig. 4A-B). To confirm the pivotal role of CAFs in directing tumor lymphangiogenesis and lymphatic metastasis *in vivo*, a nude mouse PLM model was used to simulate the directional drainage and metastasis of primary CCA to the lymph nodes. LNM was evaluated using *in vivo* imaging, gross anatomy, H&E staining, and IHC (Supplementary Fig. 12A-D). *In vivo* imaging and H&E staining showed that neither QBC939 nor HuCCT1 cells alone were able to metastasize to the PLNs after footpad inoculation. Meanwhile, at least one mouse with LNM was observed in the CAF and cancer cell co-injected groups, and the proportion of LNM was significantly increased in the mice co-injected with LN^+^CAFs (QBC939, LN^+^ CAFs *vs*. LN^-^ CAFs, 5/6 vs. 2/6; HuCCT1, LN^+^ CAFs vs LN^-^ CAFs, 4/6 vs 1/6) (Fig. 4D-J). Furthermore, IHC analyses revealed that the IMLD (LYVE1^+^ or PROX-1^+^) in the LN^+^CAF co-injection groups was higher than that in the LN^-^CAF and control groups (Fig. 4K-L, S13A). Taken together, these results suggested that LN^+^ CAFs greatly promote tube formation of LECs *in vitro*, lymphangiogenesis and LNM of CCA *in vivo*.

### PDGFB knockout in LN^+^CAFs can reduce the promotion of lymphangiogenesis and lymphatic metastasis in cholangiocarcinoma

To investigate the role of PDGF-BB in LNM, we first stimulated HLECs with PDGF-BB (VEGF-C as a positive control) (Supplementary Fig. 14A-F) and found that PDGF-BB could significantly promote HLEC migration and lymphatic vessel formation. Blocking antibodies to the VEGF-C receptor, VEGFR3, significantly inhibited VEGF-C-induced tube formation and HLEC migration, but was unable to interfere with the effect of PDGF-BB (Supplementary Fig. 14G-I), suggesting that PDGF-BB induction of lymph vessels is independent of VEGF-C. Moreover, *PDGFB* knockout LN^+^ CAFs (CAF*^PDGFB^*^(KO)^) were used to detect the role of PDGF-BB in LN^+^ CAF-induced lymphangiogenesis and LNM. We observed that knockout of *PDGFB* resulted in a decrease in the number and size of lymphatic vessels and migrated HLECs induced by the LN^+^CAF-derived supernatant (Fig. 5A-C). These findings indicated a crucial role of PDGF-BB derived from CAFs in LNM, which was further confirmed by the PLM. Although co-injection of LN^+^CAFs with CCA cells induced PLM in nearly all mice, it was almost undetectable by *in vivo* imaging or H&E staining after *PDGFB* knockout in LN^+^CAFs, except in some mice (Fig. 5D-J). In addition, IHC analyses revealed that the IMLD was decreased in the CAF*^PDGFB^*^(KO)^ group compared with that in the CAF control group (Fig. 5K-L, S13B), suggesting that CAF-derived PDGF-BB promoted LNM partly by inducing lymphangiogenesis. Taken together, these results demonstrated the necessity for PDGF-BB in LNM induced by CAFs.

### CAF-derived PDGF-BB strongly promotes the aggressiveness of CCA cells through the PDGF-BB/PDGFR-β pathway both in vitro and in vivo

To determine whether targeting the PDGF-BB/PDGFR paracrine signaling axis could inhibit tumor growth and LNM, we first assessed the effects of PDGF-BB blocking antibodies and the PDGFR inhibitor STI571 on CCA cell migration, invasion, TrEM, and clonal formation, as well as on HLEC migration and lymph vessel formation induced by CAF supernatants. Their potential inhibitory effects on tumor growth and LNM induced by LN^+^ CAFs were further investigated in mouse models (Fig. 6A, S12A). Both anti-PDGF-BB antibodies and STI571 significantly inhibited CAF-induced CCA cell migration, invasion, TrEM, and clonal formation (Supplementary Fig. 15A-C), and also reduced migration and lymph vessel formation (Fig. 7A-C). Consistent with the results of the *in vitro* experiments, the subcutaneous tumor graft volume and weight of nude mice in the anti-PDGF antibodies and STI571 groups were significantly lower than those in their control groups (Fig. 6B-G) which might be attributed to the reduced cell proliferation (Fig. 6H-K). In addition, in the PLM, anti-PDGF-BB antibodies and STI571 also inhibited LNM caused by co-injection of CAFs and CCA cells (Fig. 7D-F, S16A-C), and correspondingly, there were fewer lymphatic vessels in the pad tumors of mice with blocked PDGF-BB/PDGFR signaling (Fig. 7G-I, S13C, S16D-F). These results demonstrated the potential role of the PDGF-BB/PDGFR paracrine signaling pathway as a therapeutic target for CCA.

**Figure 8.**
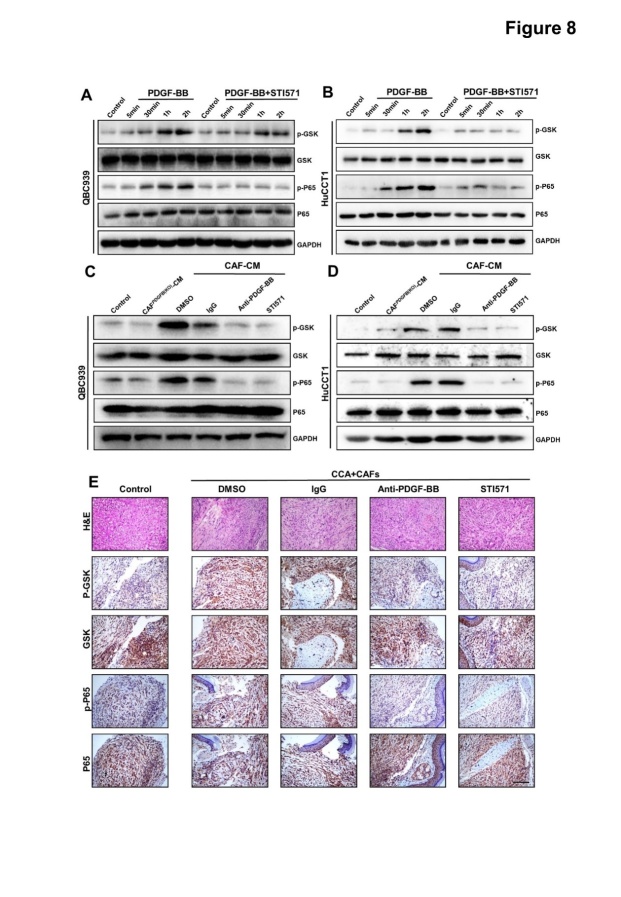
**GSK and P65 signaling pathways in CCA were activated by CAF-derived PDGF-BB through the PDGF-BB/PDGFR-β pathway**. (A, B) Representative western blotting results of GSK and P65 signaling pathway proteins in QBC939 (A) and HuCCT1 (B) cell lines treated with PDGF-BB or PDGF-BB plus STI571. (C, D) Representative western blotting results of GSK and P65 signaling pathway proteins in QBC939 (C) and HuCCT1 (D) cell lines treated with CAF supernatant treated with DMSO (0.1%), IgG (50 ng/ml), anti-PDGF-BB antibodies (50 ng/ml), or STI571(100μg/ml). (**E**) Immunohistochemical staining of GSK and P65 signaling pathways members in QBC939 tumors. Scale bar, 100 μm. n = 5 animals per group.

**Figure 9.**
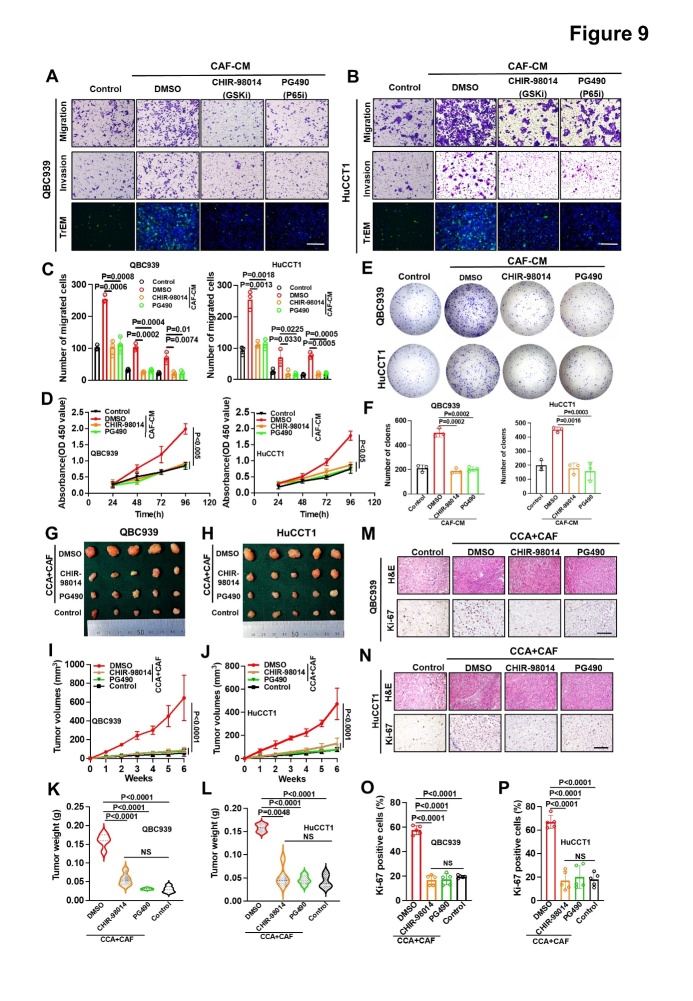
**Blocking CAF GSK and P65 signaling pathways reduced the promoting effect on CCA cells *in vitro* and *in vivo***. (A-C) Representative images (A, B) and histogram analysis (C) of the migration, invasion, and TrEM assays of QBC939 and HuCCT1 cells incubated with CAF supernatant and treated with DMSO (0.1%), CHIR-98014 (2.0μmol/L), or PG490 (100nmol/L). (**D**) MTT assays to measure the proliferation of QBC939 and HuCCT1 cells incubated with CAF supernatant treated with DMSO (0.1%), CHIR-98014 (2.0μmol/L), or PG490 (100 nmol/L). (E, F) Representative images (E) and histogram analysis (F) of QBC939 and HuCCT1 colony formation. (G, H) Representative images of QBC939 (G) and HuCCT1 (H) cells combined with CAFs on nude mouse xenografts treated with CHIR-98014 (20 mg/kg) and PG490 (1 mg/kg) (n = 5). (I, K) QBC939 tumor volumes (I) and weights (K) (n = 5). (J, L) HuCCT1 tumor volumes (J) and weights (L) (n = 5). (M-P) Representative images (M, N) and histogram analysis (O, P) of immunohistochemical staining for Ki67 expression (n = 5). All *in vitro* experiments were performed with three biological replicates or three independent experiments. The error bars indicate the SD of the mean. Statistical method: (C-D, F, K-L, O-P) One-way ANOVA, (D, I-J) Two-way ANOVA. Scale bars: (A, B) 100 μm, (M, N) 200 μm.

### CAF-derived PDGF-BB activated the GSK and P65 signaling pathways through PDGFR-β in CCA

To reveal the downstream functional signaling pathway of PDGF-BB/PDGFFRβ, we performed western blotting analysis of members of the classical downstream pathways of the PDGF-BB/PDGFFRβ signaling axis, such as AKT, JNK, ERK1/2, YAP, FAK, GSK3β, and P65, in CCA cells after treatment with or without PDGF-BB. We observed increased levels of phosphorylated GSK3β and P65 (Supplementary Fig. 17A-B), suggesting that GSK3β and P65 might be the main downstream effectors of PDGF-BB/PDGFR-β signaling in CCA cells. Moreover, the PDGFR inhibitor STI571 significantly inhibited PDGF-BB-induced activation of GSK3β and NF-κB, further demonstrating that GSK3β and P65 are the downstream effectors of PDGF BB/PDGFR-β in CCA cells (Fig. 8A-B).

To determine whether CAFs could also activate GSK3β and P65 in CCA cells through PDGF-BB/PDGFR-β, we added PDGF-BB neutralizing antibodies to the supernatant or pretreated CCA cells with a PDGFR-β inhibitor. The results showed that the CAF supernatant, but not the CAF*^PDGFB^*^(KO)^ supernatant, significantly activated GSK3β and P65 signaling, which could be weakened by PDGF-BB blockade and PDGFR inhibition (Fig. 8C-D), suggesting that CAFs activated the GSK3β and P65 pathways in CCA cells through the secretion of PDGF-BB. Furthermore, we performed IHC staining to detect the activation of AKT, JNK, ERK1/2, YAP, FAK, GSK3β, and P65 in the subcutaneous tumors from mice used in Fig. 6B. As expected, the levels of p-GSK and p-P65 were significantly reduced after treatment with anti-PDGF-BB and STI571 (Fig. 8E), while the levels of p-JNK, p-ERK1/2, p-FAK, p-AKT, and p-YAP were not different among the groups (Supplementary Fig. 18). These results indicated that CAF-derived PDGF-BB promotes CCA progression by activating GSK3β and P65 signaling downstream of PDGFR-β.

### Blocking the GSK and P65 signaling pathways of CAFs can reduce their promoting effects on CCA cells in vitro and in vivo

We next sought to explore the functional effect of inhibiting the GSK3β and P65 signaling pathways induced by PDGF-BB or CAF supernatant on CCA cells *in vitro* and *in vivo*. The inhibitors of the GSK3β (CHIR-98014) and P65 (PG490) only inhibited the activation of their respective pathways, but did not affect the other pathway (Supplementary Fig. 19A-B), indicating that the two pathways were located downstream of PDGF-BB/ PDGFR-β, without interference. We observed that the promoting effects of CAFs on the proliferation, migration, invasion, TrEM, and clonal formation of HuCCT1 and QBC939 cells were weakened under treatment by CHIR-98014 and PG490 (Fig. 9A-F), which significantly prevented CAFs from promoting tumor growth *in vivo*, both in terms of volume and weight (Fig. 9G-I). This preventive effect might be related to a reduction in cell proliferation levels (Fig. 9M-P). We also tested the inhibitory effects of CHIR-98014 and PG490 on the activation of GSK3β and P65 signaling pathways in tumors from the mouse model. The p-GSK and p-P65 levels were significantly reduced in the CHIR-98014 and PG490 groups compared with those in their respective controls. Consistent with the *in vitro* findings, the GSK3β and P65 signaling pathways did not regulate each other (Supplementary Fig. 19C-D). Taken together, these results indicated that targeting the GSK3β and P65 pathways might reduce the promoting effects of CAFs on the malignant degeneration of CCA cells *in vitro* and *in vivo*.

**Figure 10.**
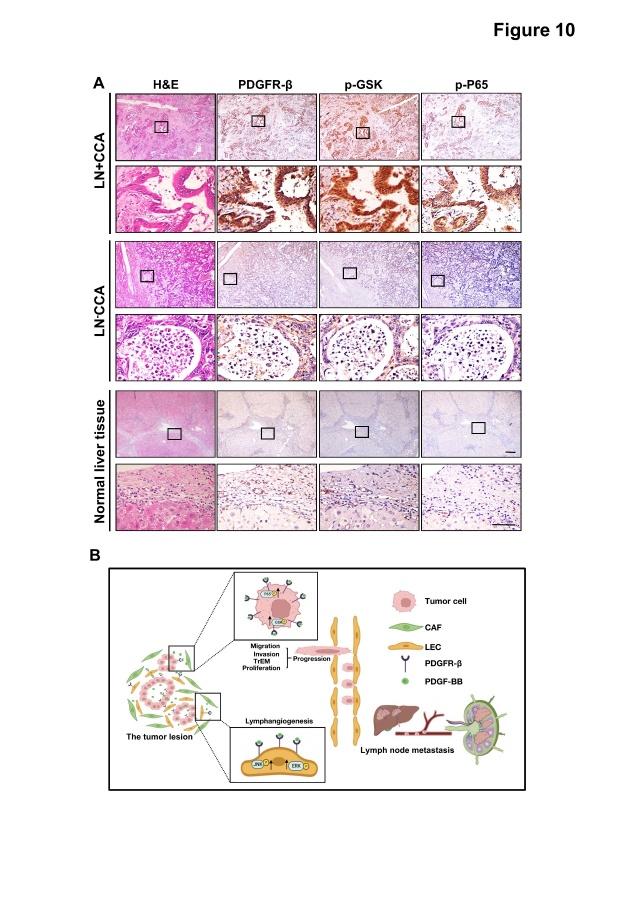
**Clinical correlations between PDGFR-β, p-GSK, and p-P65 in CCA tissues**. (**A**) PDGFR-β, p-GSK, and p-P65 levels in human CCA specimens. Bars: 100 μm. (**B**) Schematic diagram representing the role of cancer-associated fibroblast-derived PDGF-BB on tumor progression and lymphatic metastasis in cholangiocarcinoma. PDGF-BB could activate the ERK1/2 and JNK signaling pathways in LECs and the GSK and P65 signaling pathways in tumor cells through PDGFR-β.

Next, we validated the correlation between PDGFR-β expression and GSK3β and P65 signaling in normal and paired tumor tissues from patients with CCA with different node statuses. IHC analysis of continuous sections showed that PDGFR-β, p-GSK, p-P65 were co-expressed in cancer cells, and showed especially high levels in tumors from patients with LN^+^CCA (Fig. 10A). Given the significant inhibitory effect of the GSK3β and P65 inhibitors on tumor growth and LNM in CCA animal studies, we hypothesized that the PDGF-BB/PDGFR-β-GSK3β/P65 axis is a potential therapeutic target in CCA (Fig. 10B).

### Activation of intracellular signaling pathways of HLECs

Finally, we explored the downstream signaling pathways activated by PDGF-BB in HLECs. PDGF-BB stimulated significant activation of JNK and ERK1/2, while the activities of AKT, YAP, FAK, GSK3β, and P65 were unchanged (Supplementary Fig. 20A). Moreover, the PDGF-BB-enhanced the levels of phosphorylated JNK and ERK1/2 were suppressed by STI571 (Supplementary Fig. 20B). In addition, the CAF supernatant, but not the CAF*^PDGFB^*^(KO)^ supernatant, increased the levels of phosphorylated JNK and ERK1/2, which were suppressed by PDGF-BB neutralizing antibodies and the PDGFR inhibitor (Supplementary Fig. 20C). In summary, our results suggested that CAFs activated the JNK and ERK1/2 pathways in HLECs via PDGF-BB/PDGFR-β signaling, and that JNK and ERK1/2 might be functional downstream signaling pathways of PDGF-BB/PDGFR-β in the regulation of HLECs by CAFs.

## DISCUSSION

Increased tumor size and metastasis, especially LNM, are risk factors for CCA progression. The complex CCA TME is populated by a variety of mesenchymal cells that interact with each other, or with cancer cells, to regulate tumor progression. Mesenchymal cells, particularly CAFs, might play important roles in CCA progression; however, the exact mechanism is poorly understood. In the current study, we revealed that CAFs promote LNM of CCA by secreting PDGF-BB and acting on PDGFR-β-expressing LECs and cancer cells, thus leading to increased intratumoral lymphatic vessel density and cancer cell malignancy, such as enhanced proliferation and TrEM abilities.

Distant metastasis of primary cancer cells is a multi-step process, which mainly includes: (1) Invading vascular vessels, such as blood vessels and lymphatic vessels; (2) metastases along the vessel; (3) settling in distal tissues and organs; and (4) colonization and amplification [16]. In these processes, different cells and various cytokines in the TME play a critical role [23-26]. Members of the PDGF family have been widely reported to be involved in the progression of multiple tumors [27-29]. In CCA, PDGF-DD plays a crucial role in LNM [30]. Although PDGF-BB is able to regulate Hedgehog survival signaling [17], it remains unclear whether it has a similar function to PDGF-DD in regulating LNM. From these studies, we noted that the cellular origins of PDGF-DD and PDGF-BB are different, with the former being derived primarily from cancer cells and the latter being derived from CAFs. In the current study, through bioinformatic analysis and IHC staining validation, we found that PDGF-BB was overexpressed in CCA, and multiple immunofluorescence staining analyses revealed that PDGF-BB and the CAF marker α-SMA were mostly co-localized, further confirming that PDGF-BB was mainly expressed in CAFs in CCA.

Furthermore, we observed that PDGF-BB levels correlated with LNM and poor CCA outcomes using clinical feature correlation analysis. There are two cognate receptors, PDGFR-α and -β [31-32]. In this study, we identified that PDGFR-β was the primary functional receptor of PDGF-BB, because the only other receptor of PDGF-BB, PDGFR-α, was seldom expressed in CCA, which was consistent with previous studies [17,33]. PDGFR-β is mainly expressed in cancer cells and LECs; therefore, we speculated that PDGF-BB might exert its function mainly through regulating the biological characteristics of cancer cells and LECs. *In vitro* experiments have shown that PDGF-BB promotes migration, invasion, clonal formation, proliferation, and TrEM of CCA cells, as well as inducing the formation of lymphatic vessels by LECs. We also revealed that CAF supernatants have the same function as PDGF-BB, in that the LN^+^ CAF supernatant has a higher potency than the LN^-^ CAF supernatant, and that this strong potency was significantly reduced for LN^+^CAFs after CRISPR/CAS9 knockout of *PDGFB*. Western blotting and ELISA analyses both confirmed that LN^+^CAFs express higher levels of PDGF-BB than LN^-^CAFs, and MS analysis of supernatants from both CAFs indicated that PDGF-BB is one of the most significantly increased cytokines in LN^+^CAFs. This suggested that PDGF-BB is crucial in LNM induced by LN^+^CAFs, which was further confirmed by adding PDGF-BB blocking antibodies and the PDGFR-β inhibitor.

We noted that in the subcutaneous tumor model, both the anti-PDGF-BB antibodies and PDGFR inhibitor STI571 significantly inhibited tumor growth in the CAF co-injection group, reducing the tumor size and weight to the same levels as in the tumor cell-only injection group. This might be related to the suppression of cell proliferation. Moreover, without exception, LNM did not occur in the PLM models in which tumor cells were inoculated in the footpad alone. LNM occurred in the majority of mice in the CAF co-injection group, while in all but a few mice, it did not occur in the anti-PDGF-BB antibodies and STI571 treatment groups. Moreover, lymph node status was consistent with the density of lymph vessels in the mice's foot pads. These results suggested that CAFs are required to promote CCA growth and LNM, and the PDGF-BB/PDGFR-β signaling pathway is crucial for CAFs to exert their promoting role, demonstrating their potential as a therapeutic target.

Lymphangiogenesis is mostly associated with the activation of VEGFR3 signaling in tumors, with VEGF-C and VEGF-D being the classical induction factors [34-36]. In this study, our results suggest that PDGF-BB induces lymphangiogenesis through a mechanism independent of the VEGFR3 signaling. By screening the classical downstream signaling pathway of PDGF-BB, we found that PDGF-BB mainly activated the GSK3β and P65 signaling pathways in tumor cells. We noted that while PDGF-BB could upregulate the phosphorylation level of YAP, the total amount of YAP also changed synchronously, which is not entirely consistent with previous reports. However, PDGF-BB primarily activates the JNK and ERK1/2 signaling pathways in LECs. These results suggested that PDGF-BB-mediated activation of downstream pathways might be related to cellular properties.

Although both GSK3β and P65 are downstream effectors of PDGF-BB in CCA cells, we found that they exist independently of each other. Moreover, CHIR-98014 and PG490 could significantly inhibit the promotive effect of PDGF-BB and CAF-derived supernatant on the malignant functional phenotype of CCA cells. In addition, depleting p-GSK and p-P65 levels could inhibit the growth of subcutaneous tumors, which is associated with tumor proliferation, in the group co-injected with CAFs and cancer cells. Interestingly, the inhibitory effect of both inhibitors on tumor growth was approximately at the level of the cancer cell-only inoculation group. These results indicated that the activation of GSK3β and P65 downstream of PDGF-BB in CCA does not have functional redundancy; however, how they mediate the growth regulation of CCA remains to be clarified. In addition, we observed that anti-PDGF-BB antibodies and STI571 could significantly reduce the levels of p-GSK3β and p-P65 in tumor tissue in the subcutaneous tumor model, mainly occurring in tumor cells. However, the change was less pronounced for p-JNK and p-ERK1/2. Therefore, we did not further investigate the effect of JNK and ERK1/2 inhibitors on LNM in the PLM model; nonetheless, this is an issue worth exploring in the future.

STI571, also known as imatinib, is an anti-tumor drug already approved by the FDA for the treatment of chronic myeloid leukemia [37-39]. In clinical trials, the use of Gleevec in patients with advanced CCA was first reported by Holcombe et al. [40], but since this study was a case-by-case report, it was not possible to assess whether patients benefited from the treatment. Additionally, Roth et al. conducted a two-center Phase II study of imatinib mesylate for the palliative second-line treatment of advanced biliary tract cancer in 2011, but the study was stopped prematurely due to poor recruitment [41]. In addition, ClinicalTrials.gov lists more than 500 clinical trials involving STI571 in prostate, breast, stomach, pancreas, ovarian and other tumors that have been enrolled, initiated, terminated, or completed. In the case of CCA, there was only one trial in advanced or metastatic CCA (NCT01153750); however, the results are not available. In summary, STI571 has been tentatively discussed as a PDGFR inhibitor in both basic and clinical studies of cholangiocarcinoma, but no firm conclusions have been drawn. Given the importance of lymph node metastasis in influencing the prognosis of cholangiocarcinoma, combined with our findings, we believe that the re-introduction of STI571 into the clinical treatment of cholangiocarcinoma is a promising attempt. The *in vitro* and *in vivo* results presented in our study demonstrated the potential of STI571 to inhibit tumor growth and LNM of CCA with active PDGF-BB/PDGFR signaling.

In conclusion, our study demonstrated that CAF-derived PDGF-BB promotes tumor growth and LNM in CCA. Mechanistically, on the one hand, PDGF-BB activates the GSK3β and P65 pathways of tumor cells to enhance their transendothelial migration and malignancy. On the other hand, it can activate the JNK and ERK1/2 pathways of lymphatic endothelial cells to induce lymphangiogenesis.

## Supplementary Materials

The Supplementary data can be found online at: www.aginganddisease.org/EN/10.14336/AD.2023.0420.


