# Supplementary material for: Cancer-Associated Fibroblasts Promote Lymphatic Metastasis in Cholangiocarcinoma via the PDGF-BB/PDGFR-β Mediated Paracrine Signaling Network
Source: Aging Dis. 2024 Feb 1;15(1):369–89. doi: 10.14336/AD.2023.0420 (PMC10796099; doi:10.14336/AD.2023.0420)
Supplement: Supplementary file 1 [file AD-15-1-369-s.pdf]

SUPPLEMENTARY DATA

Cancer-Associated Fibroblasts Promote Lymphatic Metastasis in Cholangiocarcinoma *via* the PDGF-BB/PDGFR-β Mediated Paracrine Signaling Network

Jian Yan, Gang Xiao, Caini Yang, Qinqin Liu, Cui Lv, Xianhuan Yu, Ziyu Zhou, Shusheng Lin, Zhenhua Bai, Haoming Lin, Rui Zhang, Chao Liu

© 2023. Yan J et al. Published online at http://www.aginganddisease.org/EN/10.14336/AD.2023.0420

SUPPLEMENTARY DATA

Supplementary Materials and Methods

Cell lines and cell culture

The human CCA cell lines, HuCCT1, HCCC-9810, Huh28, QBC939, and the immortalized normal human intrahepatic biliary epithelial cells (HiBEpics) were purchased from Otwo Biotech (Guangzhou China). The human CCA cell line ZJU-1125 was a friendly gift from Professor Yang of Zhejiang University. CAFs isolated from CCA tumor tissues were immortalized by Applied Biological Materials Inc. (ABM, Richmond, Canada). CAFs and QBC939 cells were cultured in Dulbecco's modified Eagle's medium (DMEM) (Gibco, C11995500BT, Grand Island, NY, USA) supplemented with 10% fetal bovine serum (FBS) (Biological Industries, 04-400-1A). The HuCCT1, ZJU-1125, HCCC-9810, Huh28, and HiBEpic cells were cultured in Roswell Park Memorial Institute 1640 (RPMI 1640) medium (Gibco) supplemented with 10% FBS. The human LEC cell line, HLECs (catalog JNO-1750) were purchased from GENNIO Biological Technology (Guangzhou China) and cultured in endothelial cell medium (ECM) (Science Cell Research Laboratory, Carlsbad, CA, USA) supplemented with 10% FBS. The cells were cultured in a humidified incubator with 5% CO_2 at 37 °C.

Isolation of primary fibroblasts from CCA specimens

The tumor specimens were obtained from patients with CCA who had undergone surgical resection. The samples were coded anonymously in accordance with local ethical guidelines. The tissues were minced and placed in DMEM supplemented with 10% FBS, collagenase type I (Worthington, LS004194, 1 mg/ml), and 100 U/ml hyaluronidase, and treated at 37 °C for 8 h. The tissue was washed twice using phosphate-buffered saline (PBS), and centrifuged at 450 × *g* for 8 min. The cells in the pellet were resuspended in DMEM supplemented with 10% FBS and 100 IU/ml levofloxacin and incubated at 37 °C in a 5% CO_2 humidified environment. CD31, CD45, Vimentin, cytokeratin 19 (CK-19), FAP, fibronectin, and α-SMA expression by the CAFs was detected using immunofluorescence. Additionally, CD31, CD45, CK-19, FAP, and α-SMA were analyzed by flow cytometry (FC). Details of all antibodies are given in Supplementary Table 2. All the *in vitro* experiments were carried out using primary fibroblasts within 4–8 passages and the *in vivo* experiments were performed using immortalized CAFs.

Characterization of fibroblasts by flow cytometric analysis

Primary fibroblasts were immunostained with fluorescent pigmented antibodies in the FACS step, while an additional endothelial marker, PE/Cy7-conjugated anti-human CD31 (Biolegend, 303118), the fibroblasts marker anti-human α-SMA (Abcam, Ab7817), anti-human FAP (ThermoFisher, BMS168), immune cell marker anti-human CD45 (Abcam, Ab40763) or epithelial cell marker CK-19 (Abcam, Ab76539) were analyzed using flow cytometry. Details of all antibodies are given in Supplementary Table 2.

Immortalization of fibroblasts

The immortalized CAFs were commissioned to be created by ABM Corporation, and the procedure is described below. Primary CAFs between passage 4 and passage 6 were inoculated in 6-well culture plates and cultured in an incubator with 5% CO_2 at 37 °C. The optimal multiplicity of infection (MOI = 10) values was determined by the virus titer and preliminary experiments. Complete culture medium containing lentivirus with sufficient numbers of SV40LT transforming protein coding sequences (Applied Biological Materials Inc., Canada) was added to the 6-well plate. Two days after infection, low-density CAFs were inoculated into the 6-well plates. In addition, successive passages were performed through low-density cultures in 6-well plates, and cultures of different passages were collected for storage or validation to test the character or functional stability of the cell passages up to the 30th generation.

Western blotting

Total proteins were extracted using Radio Immunoprecipitation Assay (RIPA) buffer. The Pierce^{TM} BCA Protein Assay Kit was used to quantify the protein concentrations. The total protein was separated using SDS-polyacrylamide gel electrophoresis and transferred to polyvinylidene fluoride (PVDF) membranes, which were then blocked with 5%

© 2023. Yan J et al. Published online at http://www.aginganddisease.org/EN/10.14336/AD.2023.0420

bovine serum albumin (BSA) for 2 h and then incubated overnight at 4 °C with primary antibodies. The membranes were washed with Tris-buffered saline-Tween 20 (TBS-T) three times, and then incubated with horseradish peroxidase (HRP)-conjugated goat anti-rabbit (1:5000, Abcam, Cambridge, MA, USA) or goat anti-mouse (1:5000, Abcam) secondary antibodies at room temperature for 1 h. Immunoreactive proteins were visualized using enhanced chemiluminescence (FDbio-Dura ECL Kit, FDbio science, Hangzhou, China). Details of the antibodies used are provided in Supplementary Table 2.

Trans-well assays

Migration assay: Cells were pre-cultured with low serum medium (1% FBS) for 24 h, then resuspended to 5×10^4 cells/ml in serum-free medium and inoculated into the upper chambers of a 24-well Transwell plate with 400 µl per well containing 20,000 cells. The bottom wells were filled with 600 µl of complete medium (10% FBS). After 24 h of incubation, the cells remaining in the upper chambers were removed and the cells at the bottom of the chambers were fixed in 4% paraformaldehyde for 15 min and stained with crystal violet for 10 min. Five randomly selected microscopic fields were used to count the cells.

Invasion assay: The procedure was the same as for the *Migration assay*, except that the Transwell chamber was pre-coated with Matrigel.

Trans-endothelial migration assay (TrEM): HLECs (50,000) were seeded on 8-µm migration chambers pre-coated with 2 µg/cm^2 fibronectin. When the cells were confluent, 5×10^4 EGFP-labeled HuCCT1 or QBC939 cells were placed in the upper chamber with the endothelial monolayer. After incubation for 24 h, the cells remaining in the upper chamber were removed, and the cells at the bottom of the chamber were fixed in 4% paraformaldehyde and stained with crystal violet for 10 min. Ten images per chamber were randomly captured at 200× magnification and the EGFP$^+$ cells were counted.

HLECs tube formation assay

A 48-well plate was pre-coated with Matrigel (Biocoat, 354234) and placed in a 37 °C constant temperature cell incubator for shaping. Each well was seeded with 5000 HLECs. The control group was cultured with DMEM medium, while the experimental group was cultured with DMEM medium containing CAF supernatants. Cytokines, inhibitors, or blocking antibodies, and their corresponding controls, such as DMSO and IgG, were added according to the experimental purpose and culture was then continued for 6 h. The lymphatic vessels were photographed and quantified by measuring the number and length of intact lymphatic structures using Image J software.

Cell proliferation assay and colony Formation Assay

All cells (5000 cells/well) were cultured in 96-well cell culture plates with 5% CO_2 at 37 °C, then treated with recombinant human PDGF-BB (PeproTech, 100-14B-100), CAF supernatant or their corresponding control. The cell viability was detected at different time points. Cell viability was measured using a Cell Counting Kit-8 (CCK-8, Yishan Biotechnology, Shanghai, China). Briefly, each well was incubated with 10 µl of water-soluble formazan dye with 5% CO_2 at 37 °C for 2 h. Then, the absorbance at 450 nm was measured using a plate reader. The absorbance (optical density, OD) of the negative control was used as the blank for comparison. For the colony formation assay, cells were seeded in 6-well plates. After 7–10 days, the cells were washed twice with PBS, fixed in 4% formaldehyde for 15 min and stained with 0.1% crystal violet for 10–30 min. Colonies with more than 50 cells were counted.

CRISPR/Cas9-mediated PDGFB gene knockout

For CRISPR mediated *PDGFB* gene deletion in human CAFs, we used two guide RNA (gRNA) sequences, which were designed and synthesized by IGEbio (Guangzhou, China: gRNA 1: GACGGCACTGAAGGAGACCCT and 2: GGTGACGCTGGAAGACCACC). The gRNAs were cloned into the lentiCRISPR-copGFP vector (IGEbio). CAFs with stable Cas9 expression were obtained using CRISPRMAX™Cas9 transfection reagent (IGEbio) and hygromycin (GENVIEW, AH169-1G) resistance screening. Viruses containing two different sgRNAs were mixed together to co-infect CAFs^{CAS9} and then the transfected cells were sorted by FACS using GFP. The knockout effect on *PDGFB* was confirmed by western blotting and ELISA.

© 2023. Yan J et al. Published online at http://www.aginganddisease.org/EN/10.14336/AD.2023.0420

SUPPLEMENTARY DATA

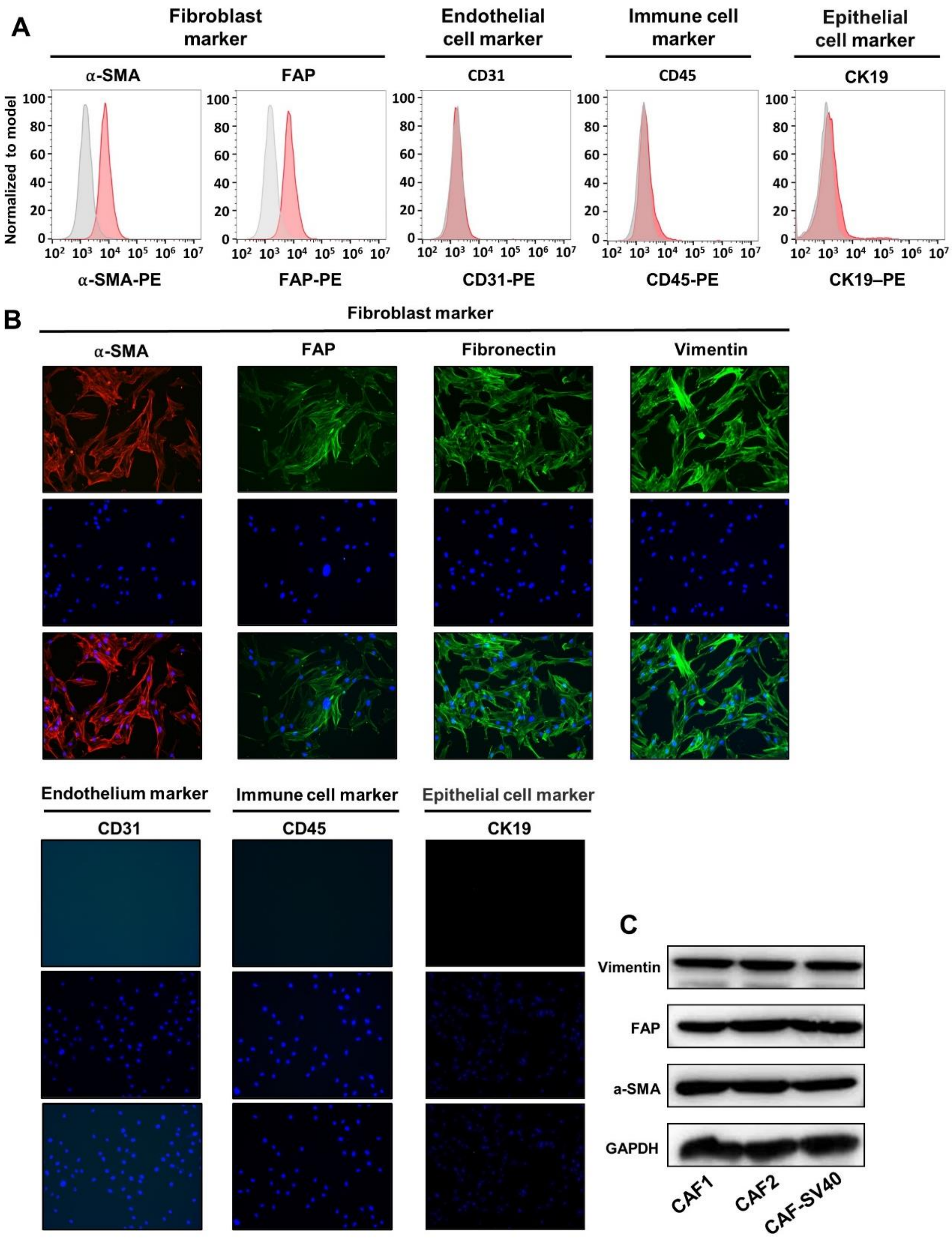

Supplementary Figure 1. Characterization of CAFs in CCA. (A) Flow cytometry analysis of CAFs isolated from patients with patients showed that the purity of fibroblast preparation was high. Fibroblast preparations displayed high expression of fibroblast markers α-SMA, FAP, and undetectable or low expression of endothelial cell marker CD31, immune cell marker CD45, and epithelial cell marker CK19. **(B)** Immunofluorescent staining analysis of markers of fibroblast, endothelial cell, immune cell, and epithelial cell in CAFs (n = 3 independent samples). **(C)** α-SMA, FAP, and vimentin expression in immortalized and non-immortalized CAFs.

© 2023. Yan J et al. Published online at http://www.aginganddisease.org/EN/10.14336/AD.2023.0420

SUPPLEMENTARY DATA

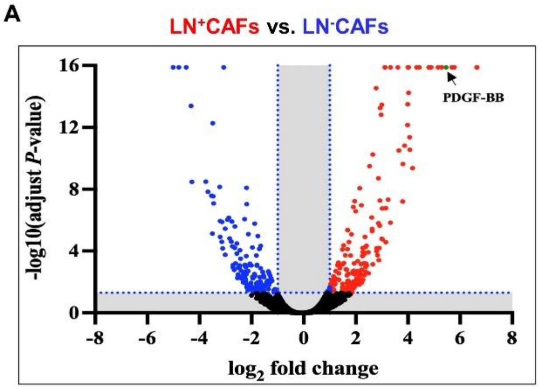

B

LN+CAFs vs. LN-CAFs

1	CYP2J2	11	ATP6V1C1
2	ARHGAP12	12	SERPINB11
3	PAN3	13	FILIP1
4	SLC4A5	14	MEP1A
5	STC1	15	STMN1
6	STAC	16	SLC25A24
7	GRID1	17	CPS1
8	**PDGFB**	18	RPL5
9	LATS2	19	GNAS
10	DHPS	20	JAG1

Supplementary Figure 2. Proteomics analysis of secreted proteins in LN⁺CAFs and LN⁻CAFs. (A) Volcano plot of the proteomic analysis showing that PDGF-BB was significantly upregulated in the LN⁺ CAF supernatant compared with the LN⁻ CAF supernatant. X-axis: The log2-fold change for each identified secretory protein from the supernatant. Y-axis: The −log10 of the p-values. Red dots indicate significantly upregulated proteins; green dots indicate significantly downregulated proteins ($p < 0.05$, fold change > 2 [log2 = 1]). Horizontal dotted line: original p = 0.05. Dots with fold change < 2 (log2 = 1) and/or $p < 0.05$ are shown in gray. Statistical tests were two-sided. **(B)** Table 2 shows the top 20 secreted proteins in LN⁺CAF supernatants compared with LN⁻ CAF supernatants.

© 2023. Yan J et al. Published online at http://www.aginganddisease.org/EN/10.14336/AD.2023.0420

SUPPLEMENTARY DATA

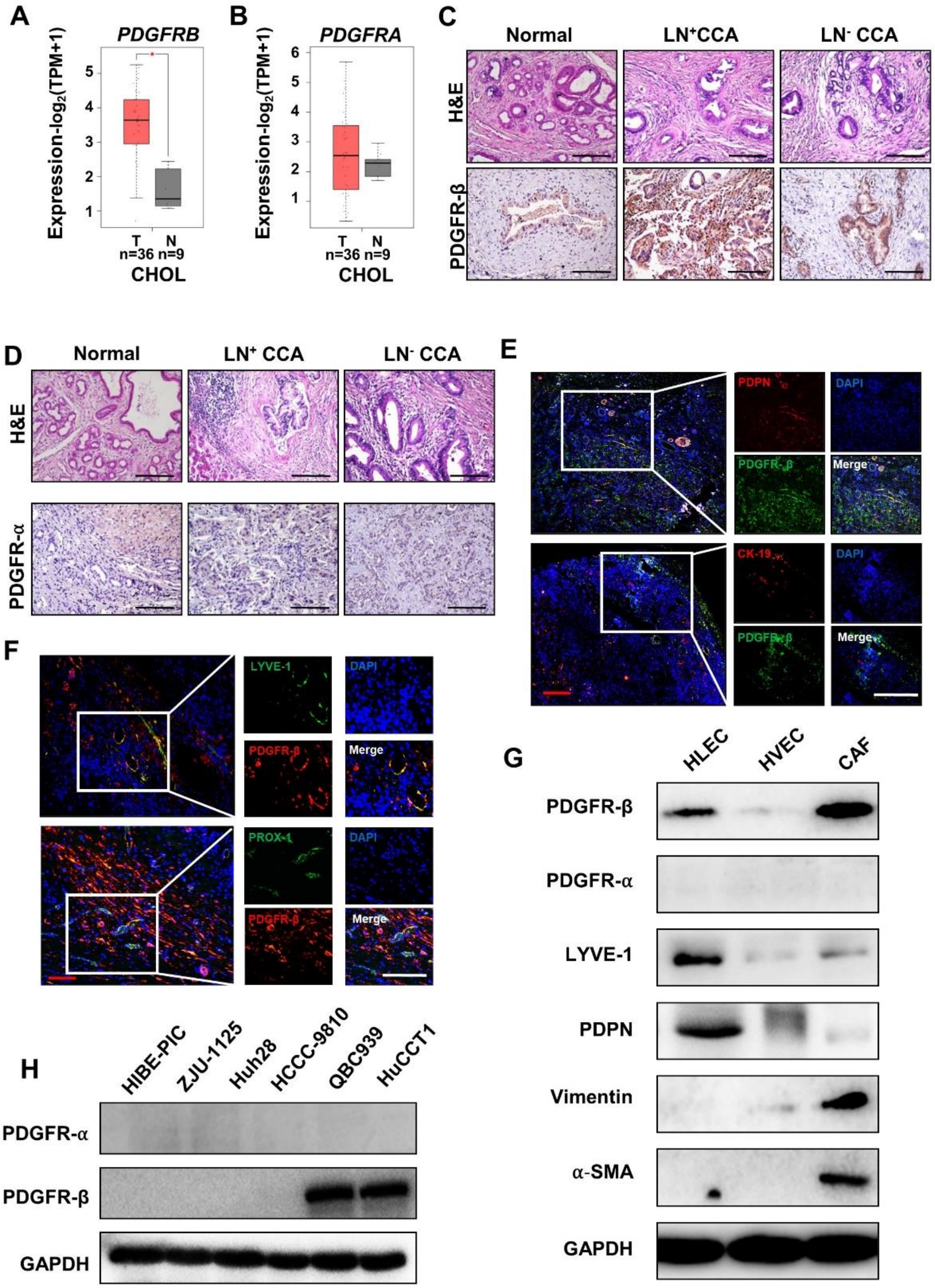

Supplementary Figure 3. The expression of PDGFR-β in CCA. (A, B) GEPIA database analysis of the relative *PDGFRB* **(A)** and *PDGFRA* **(B)** mRNA expression in matched CCA tissues and adjacent non-CCA tissues. **(C)** Representative images of PDGFR-β immunohistochemical staining in human LN⁺CCA, LN⁻CCA, and non-cancer tissues. **(D)** Representative images of PDGFR-α immunohistochemical staining in human LN⁺CCA, LN⁻CCA, and non-cancer tissues. **(E)** Representative images of PDGFR-β/PDPN (TSA) and PDGFR-β/CK-19 double-labeled immunofluorescence staining in cholangiocarcinoma. **(F)** Representative images of PDGFR-β/LYVE-1 and PDGFR-β/PROX-1 double-labeled immunofluorescence staining in cholangiocarcinoma (TSA). **(G)** The expression of different phenotypic markers in HLEC, HVEC and CAFs. **(H)** The intracellular PDGFR-α and PDGFR-β protein levels in CCA cell lines and HIBE-PICs, respectively. Scale bars: (C-D) 200 μm, (E-F) red, 50 μm, white 100 μm.

© 2023. Yan J et al. Published online at http://www.aginganddisease.org/EN/10.14336/AD.2023.0420

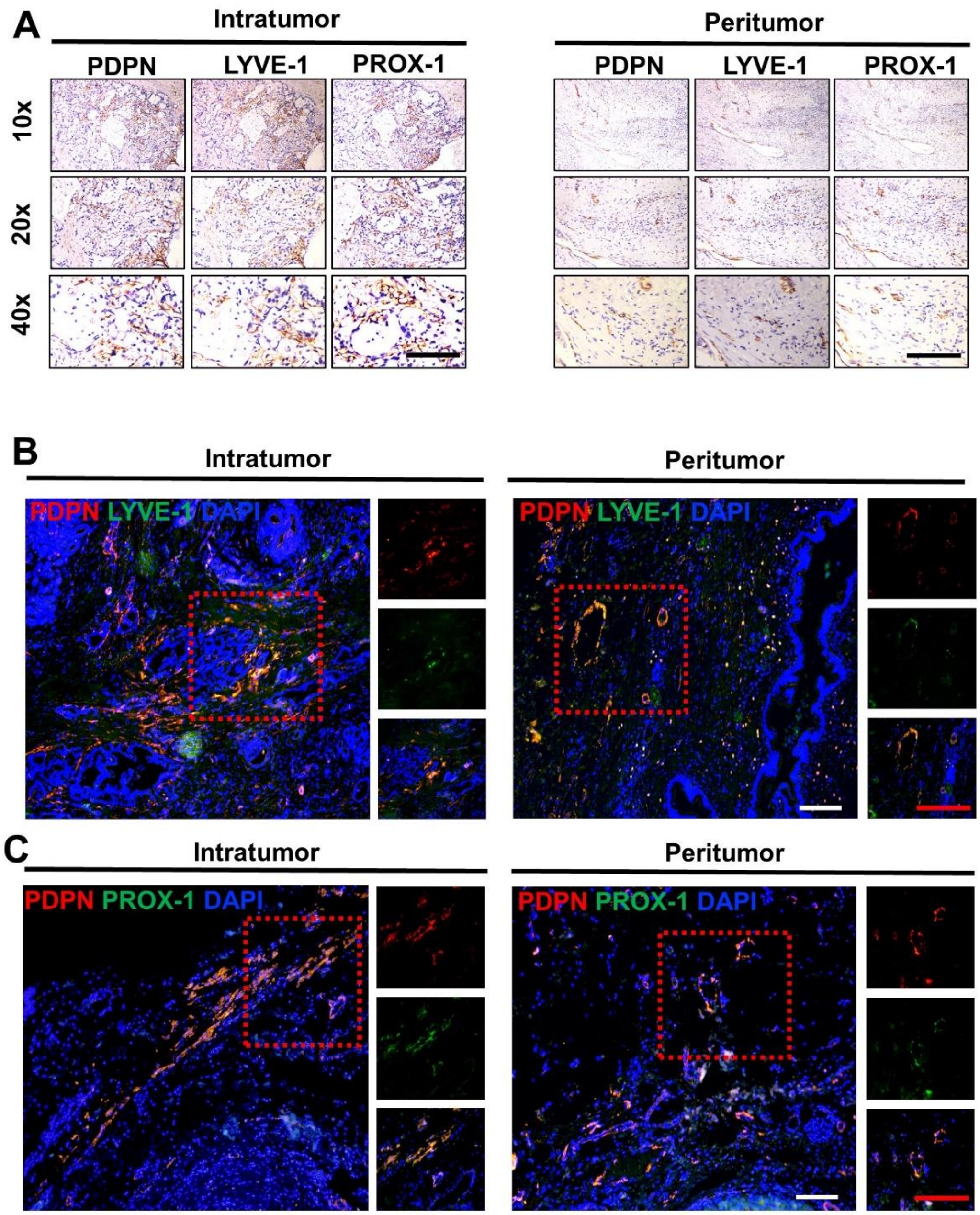

Supplementary Figure 4. Markers of lymphoendothelial cells in cholangiocarcinoma. (A) Representative images of immunohistochemical staining in continuous paraffin sections for PDPN, LYVE-1, and Prox-1 in intratumoral and peritumoral tissues of cholangiocarcinoma. **(B)** Representative images of PDPN/LYVE-1 double-labeled immunofluorescence staining in intratumoral and peritumoral tissues of cholangiocarcinoma (TSA). **(C)** Representative images of PDPN/PROX-1 double-labeled immunofluorescence staining in intratumoral and peritumoral tissues of cholangiocarcinoma (TSA).

© 2023. Yan J et al. Published online at http://www.aginganddisease.org/EN/10.14336/AD.2023.0420

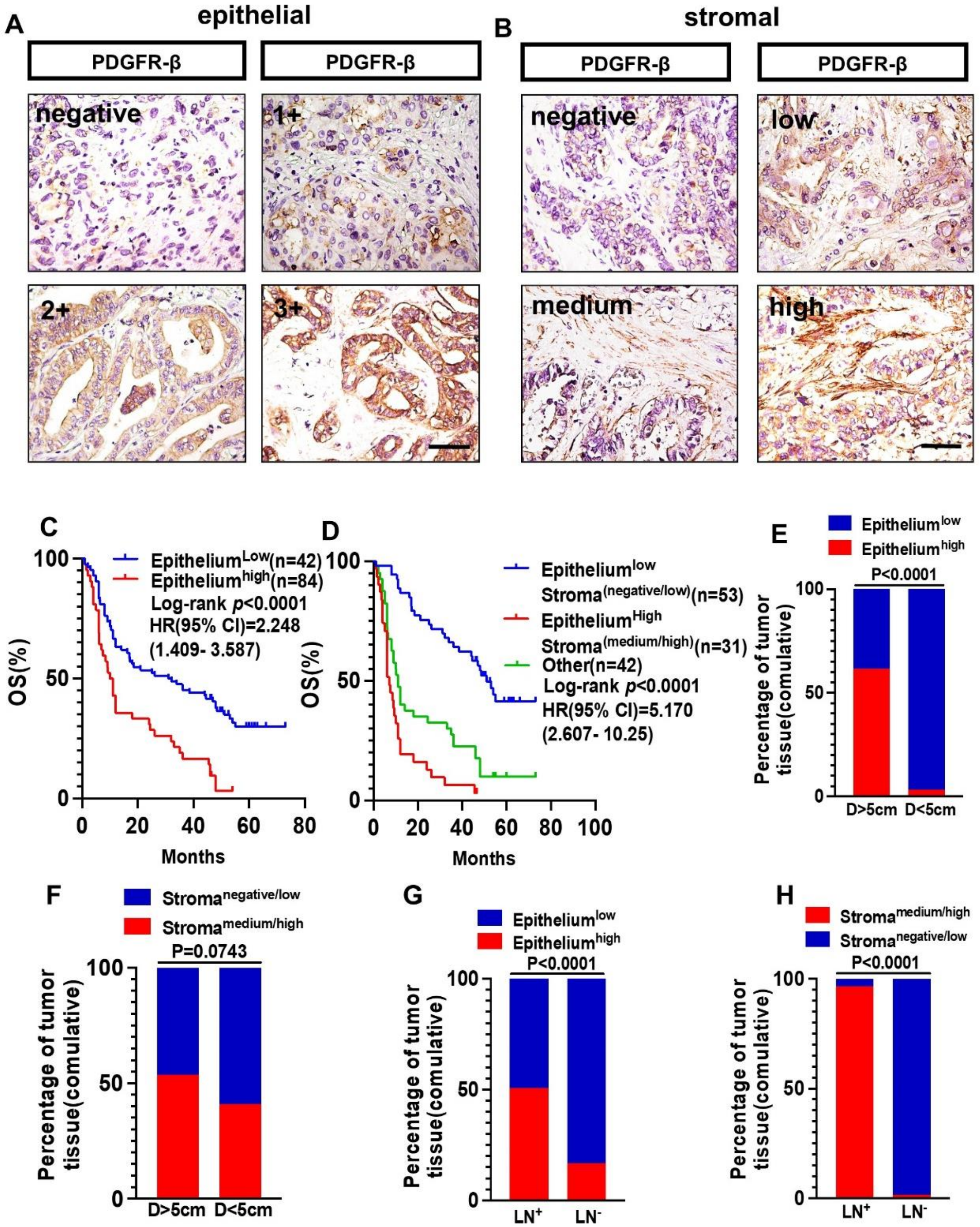

Supplementary Figure 5. The expression of PDGFR-β correlated with LNM in CCA. (A) Scoring scheme for immunostaining of epithelium (negative to 3+) and **(B)** stromal (negative–high) expression of PDGFR-β in cholangiocarcinoma. Appropriate validation on > 10 independent samples was performed to ensure reproducible staining patterns in CCA tissue. **(C, D)** Human CCA tissues were classified into epithelium^{High} or epithelium^{Low} groups, **(C)** or epithelium^{High}/Stroma$^{Medium/High}$ and epithelium^{Low}/Stroma$^{Negative/Low}$ groups **(D)** based on PDGFR-β IHC, followed by examining patient overall survival using Kaplan–Meier survival analysis. **(E)** The percentages of the PDGFR-β epithelium^{High} or epithelium^{Low} expression in the CCA tissues with diameters > 5 cm or < 5 cm. **(F)** The percentages of the PDGFR-β Stroma$^{Medium/High}$ or Stroma$^{Negative/Low}$ expression in the CCA tissues with diameters > 5 cm or < 5 cm. **(G)** The percentages of the PDGFR-β epithelium^{High} or epithelium^{Low} expression in the CCA tissues with or without LN metastasis. **(H)** The percentages of the PDGFR-β Stroma$^{Medium/High}$ or Stroma$^{Negative/Low}$ expression in the CCA tissues with or without LN metastasis. Statistical method: (E-F) χ^2 test. Scale bar, 200 μm.

© 2023. Yan J et al. Published online at http://www.aginganddisease.org/EN/10.14336/AD.2023.0420

SUPPLEMENTARY DATA

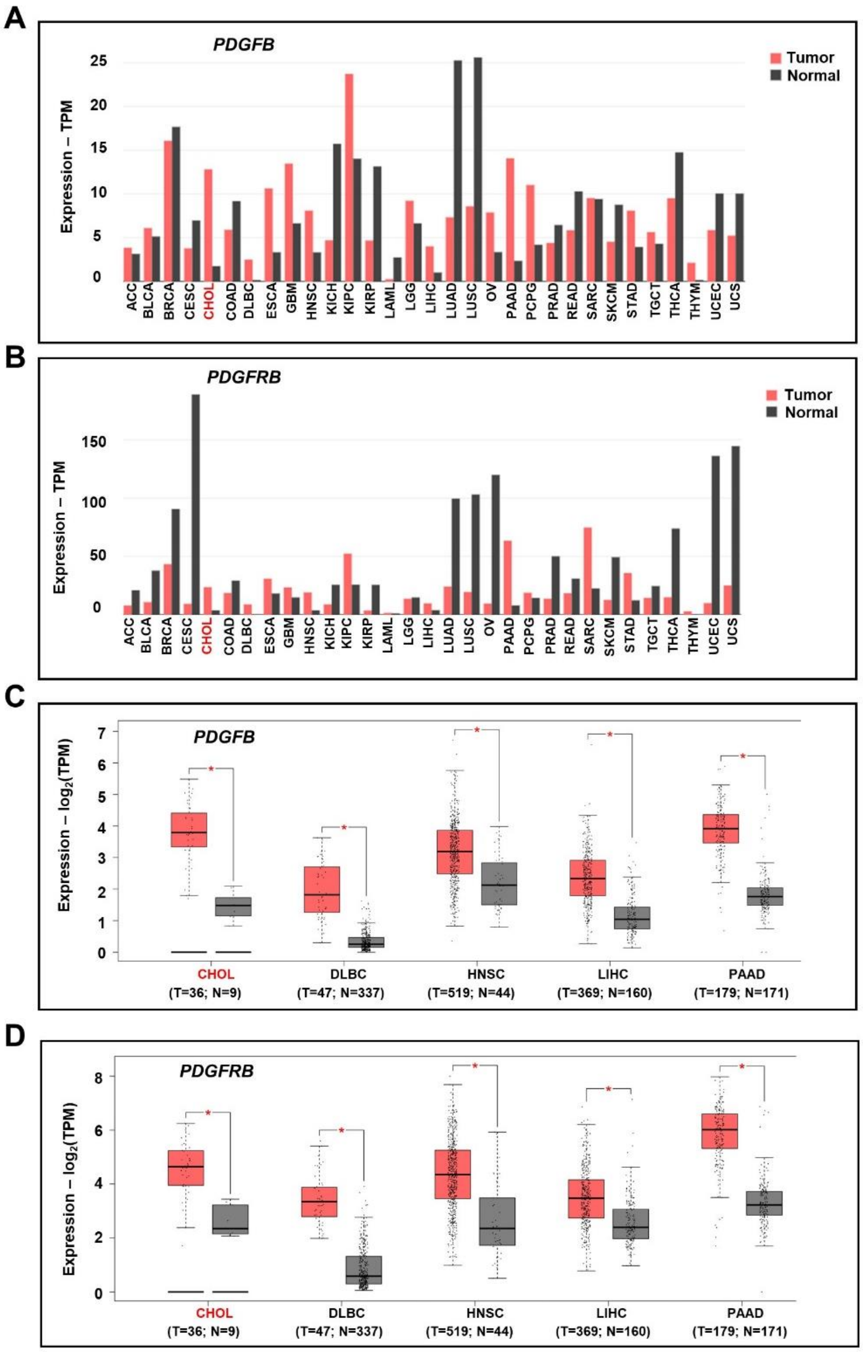

Supplementary Figure 6. The GEPIA data of *PDGFB* and *PDGFRB* expression in human tumors. (A, B) The GEPIA data for *PDGFB* **(A)** and *PDGFRB* **(B)** expression in human tumors. *PDGFB* was significantly highly expressed in Cholangiocarcinoma (CHOL), Lymphoid Neoplasm Diffuse Large B-cell Lymphoma (DLBC), Esophageal carcinoma (ESCA), Glioblastoma multiforme (GBM), Head and Neck squamous cell carcinoma (HNSC), Liver hepatocellular carcinoma (LIHC), Ovarian serous cystadenocarcinoma (OV), Pancreatic adenocarcinoma (PAAD), Pheochromocytoma and Paraganglioma (PCPG), Stomach adenocarcinoma (STAD), and Thymoma (THYM). PDGFR-β was significantly highly expressed in CHOL, DLBC, HNSC, LIHC, Kidney renal clear cell carcinoma (KIRC), PAAD, and Sarcoma (SARC). **(C, D)** Both *PDGFB* **(C)** and *PDGFRB* **(D)** were highly expressed in CHOL, DLBC, HNSC, LIHC, and PAAD.

© 2023. Yan J et al. Published online at http://www.aginganddisease.org/EN/10.14336/AD.2023.0420

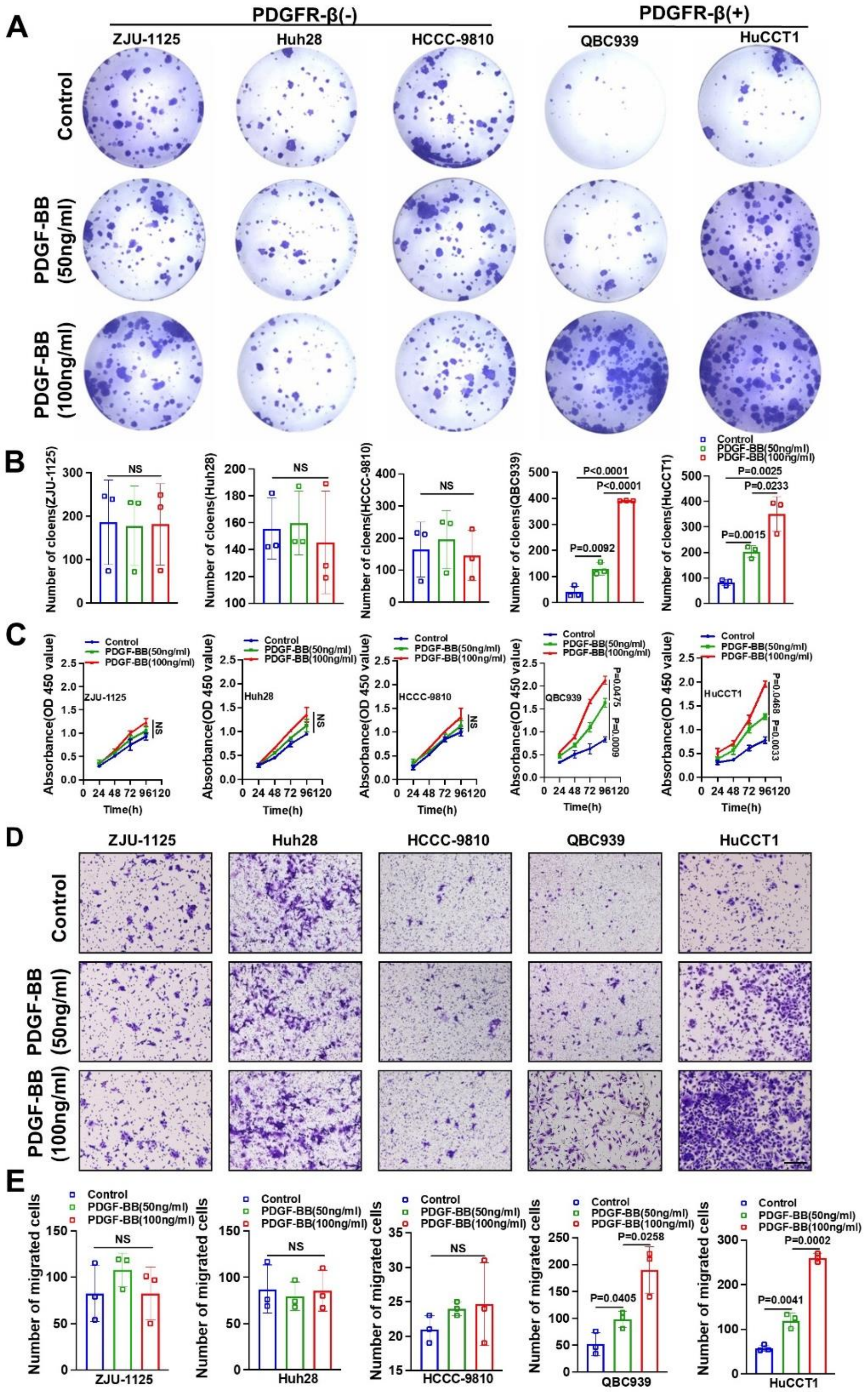

Supplementary Figure 7. PDGF-BB promotes proliferation, colony-forming ability, migration of CCA cells *in vitro*. (A, B) Representative images **(A)** and quantification **(B)** of colony formation for PDGFR-β negative cells (ZJU-1125, Huh28, HCCC-9810) and PDGFR-β positive cells (QBC939, HuCCT1) treated with PDGF-BB at different concentrations. **(C)** Cell proliferation abilities for PDGFR-β negative cells (ZJU-1125, Huh28, HCCC-9810) and PDGFR-β positive cells (QBC939, HuCCT1) treated with PDGF-BB at different concentrations. **(D, E)** Representative images of the cell migration assay for PDGFR-β negative cells (ZJU-1125, Huh28, HCCC-9810) and PDGFR-β positive cells (QBC939, HuCCT1) treated with PDGF-BB at different concentrations. All *in vitro* experiments were performed with three biological replicates or three independent experiments. The error bars indicate the SD of the mean. Statistical method: (B, E) One-way ANOVA, (C) Two-way ANOVA. Scale bars: 100 μm.

© 2023. Yan J et al. Published online at http://www.aginganddisease.org/EN/10.14336/AD.2023.0420

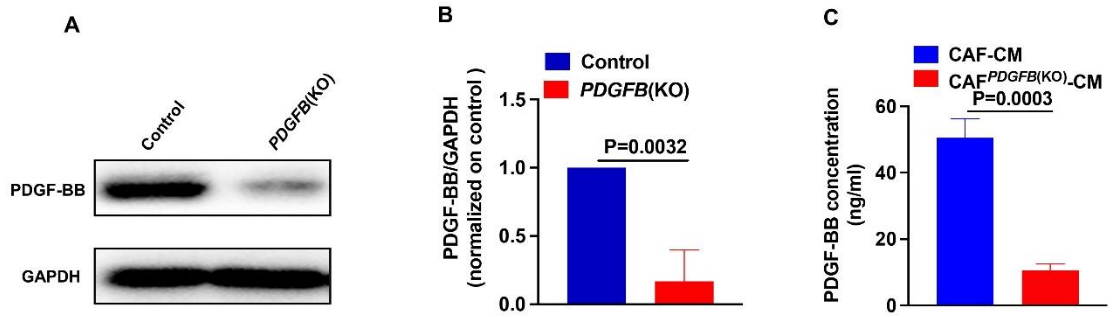

Supplementary Figure 8. ***PDGFB*** **knockout in CAFs. (A-C)** Stable knockout of *PDGFB* in CAFs was confirmed by western blotting **(A, B)** and ELISA **(C)**. The results are representative of three independent experiments and are shown as the mean ± SD. Statistical method: (B, C) Student's t test.

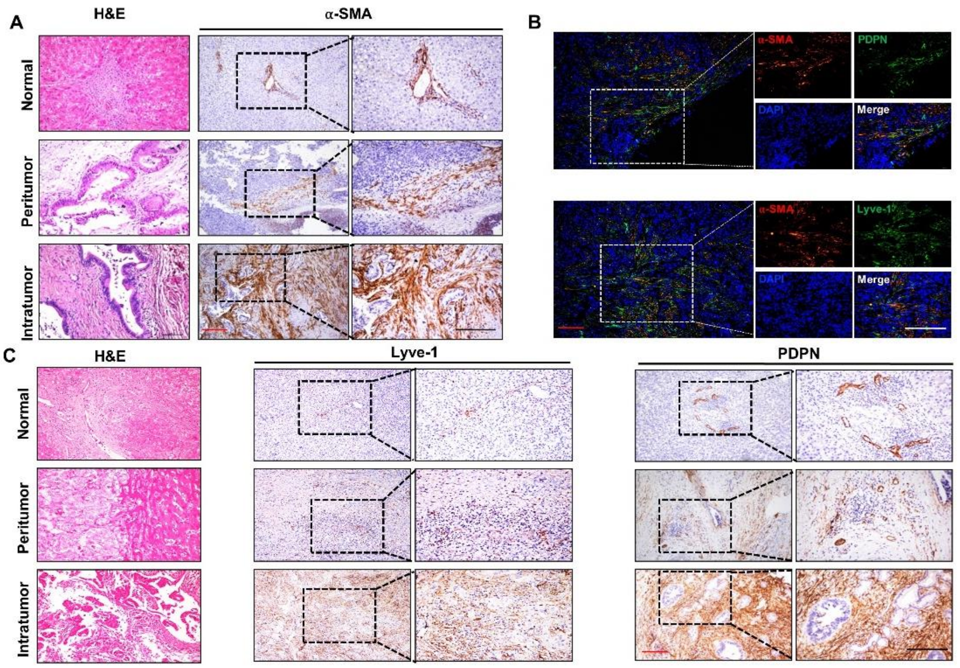

Supplementary Figure 9. CCA tissues demonstrated more CAFs aggregation and higher LMVD than normal liver tissues. (A) The density of CAFs was more higher in intratumoral tissue than in peritumoral tissue of CCA and normal liver tissue, as shown by representative immunohistochemical staining images for α-SMA. **(B)** LECs were localized close to CAFs in the CCA stroma, as shown by double immunofluorescence of PDPN, LYVE1 (green), and α-SMA (red). **(C)** LMVD expression was higher in intratumoral tissue than in peritumoral tissue of CCA and normal liver tissue, as shown by representative immunohistochemistry staining images for PDPN and LYVE1. Scale bars: (A, C) red, 50 µm, black, 100µm, (B) red, 50 µm, white 100µm.

© 2023. Yan J et al. Published online at http://www.aginganddisease.org/EN/10.14336/AD.2023.0420

SUPPLEMENTARY DATA

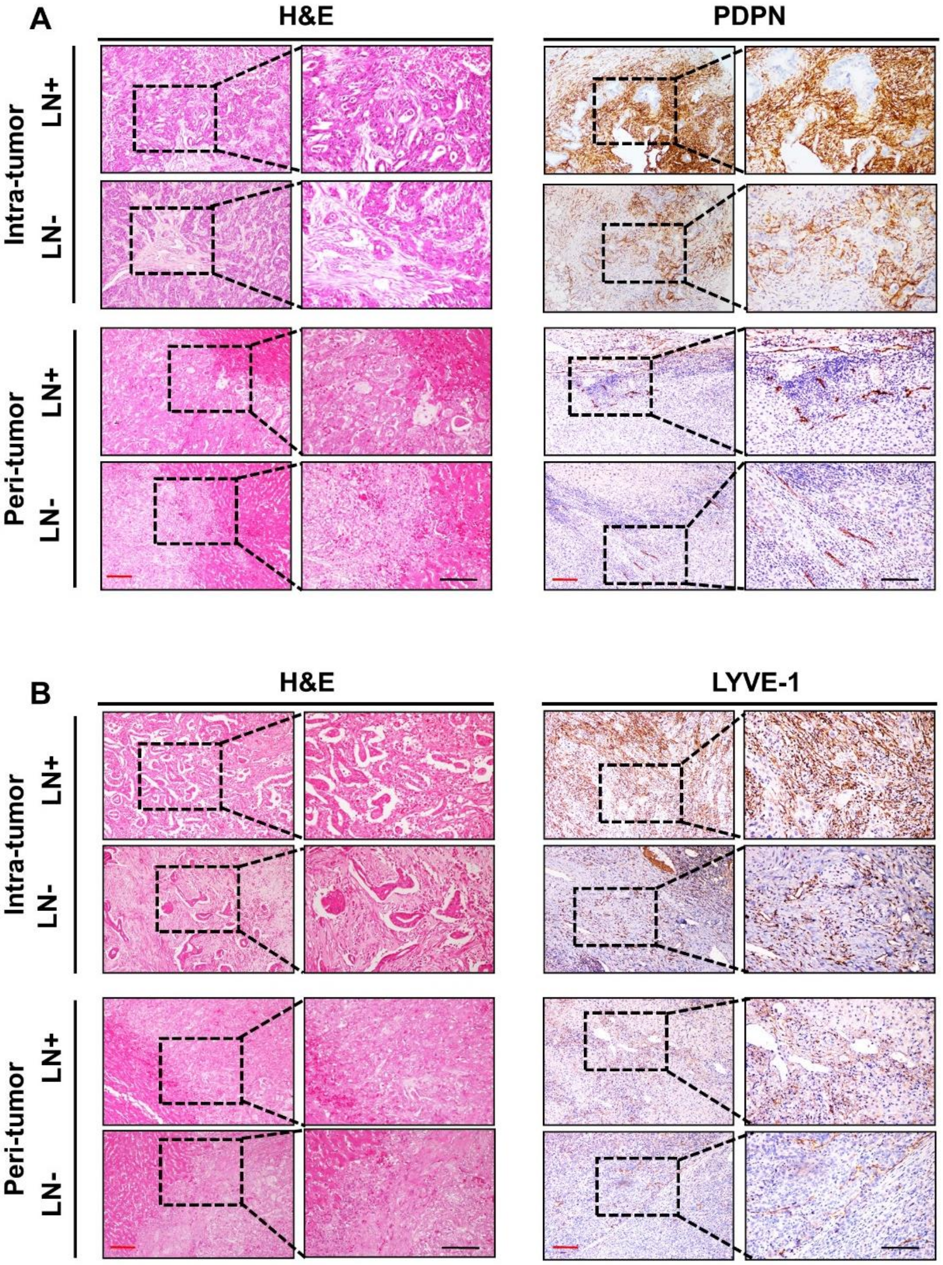

Supplementary Figure 10. Immunohistochemical staining of LMVD in CCA tissues. (A, B) LMVD expression was higher in LN^+ CCA tissues than in LN^- CCA tissues, as shown by representative immunohistochemical staining images for PDPN **(A)**, and LYVE1 **(B)**. Scale bars: red, 50 μm, black, 100μm.

© 2023. Yan J et al. Published online at http://www.aginganddisease.org/EN/10.14336/AD.2023.0420

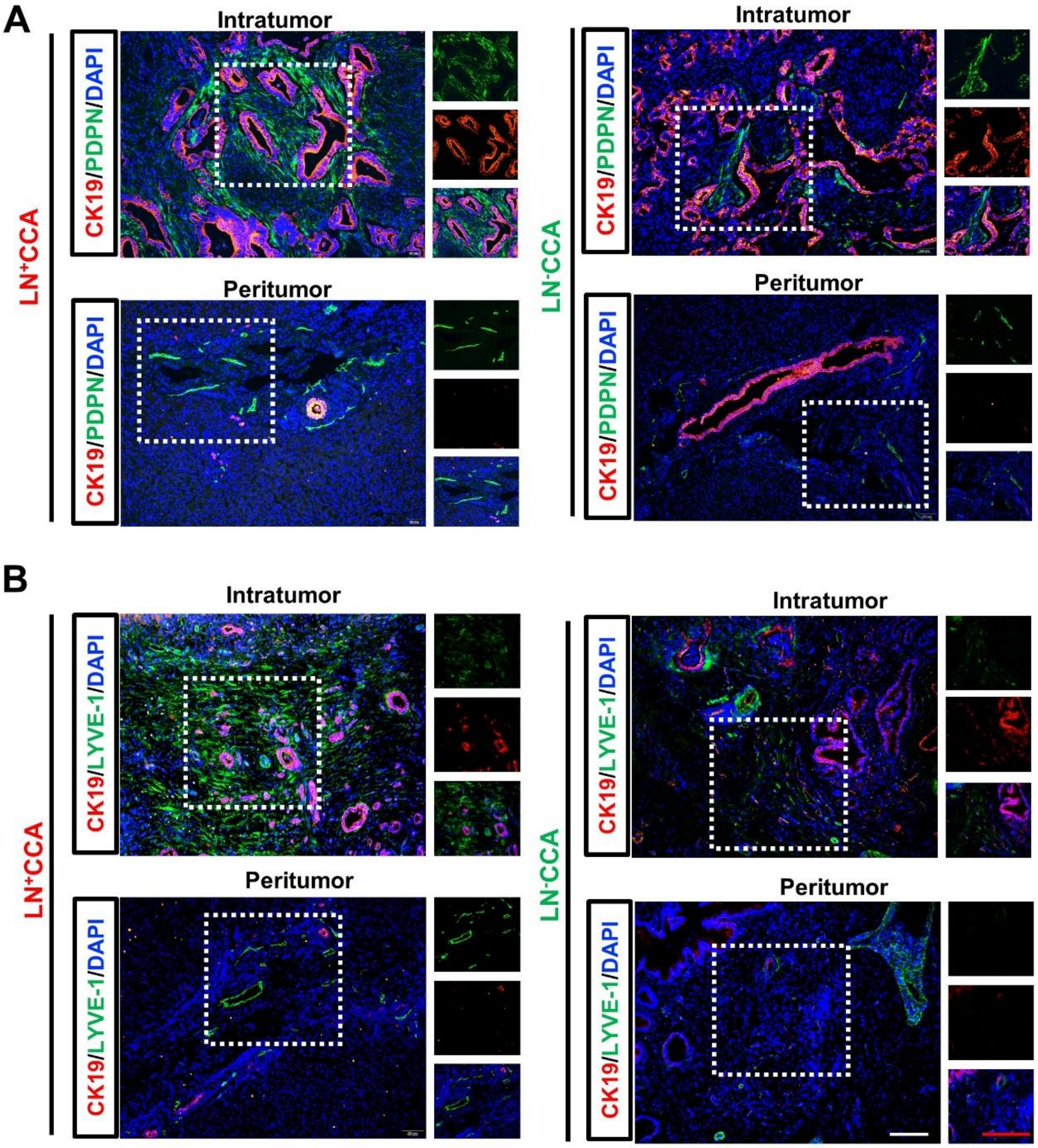

Supplementary Figure 11. Immunofluorescence staining of LMVD in CCA tissues. (A) Representative images of CK-19/PDPN double-labeled immunofluorescence staining in intratumoral and peritumoral tissues of cholangiocarcinoma. **(B)** Representative images of CK-19/LYVE-1 double-labeled immunofluorescence staining in intratumoral and peritumoral tissues of cholangiocarcinoma. Scale bars: white, 50 µm, red, 100µm.

© 2023. Yan J et al. Published online at http://www.aginganddisease.org/EN/10.14336/AD.2023.0420

SUPPLEMENTARY DATA

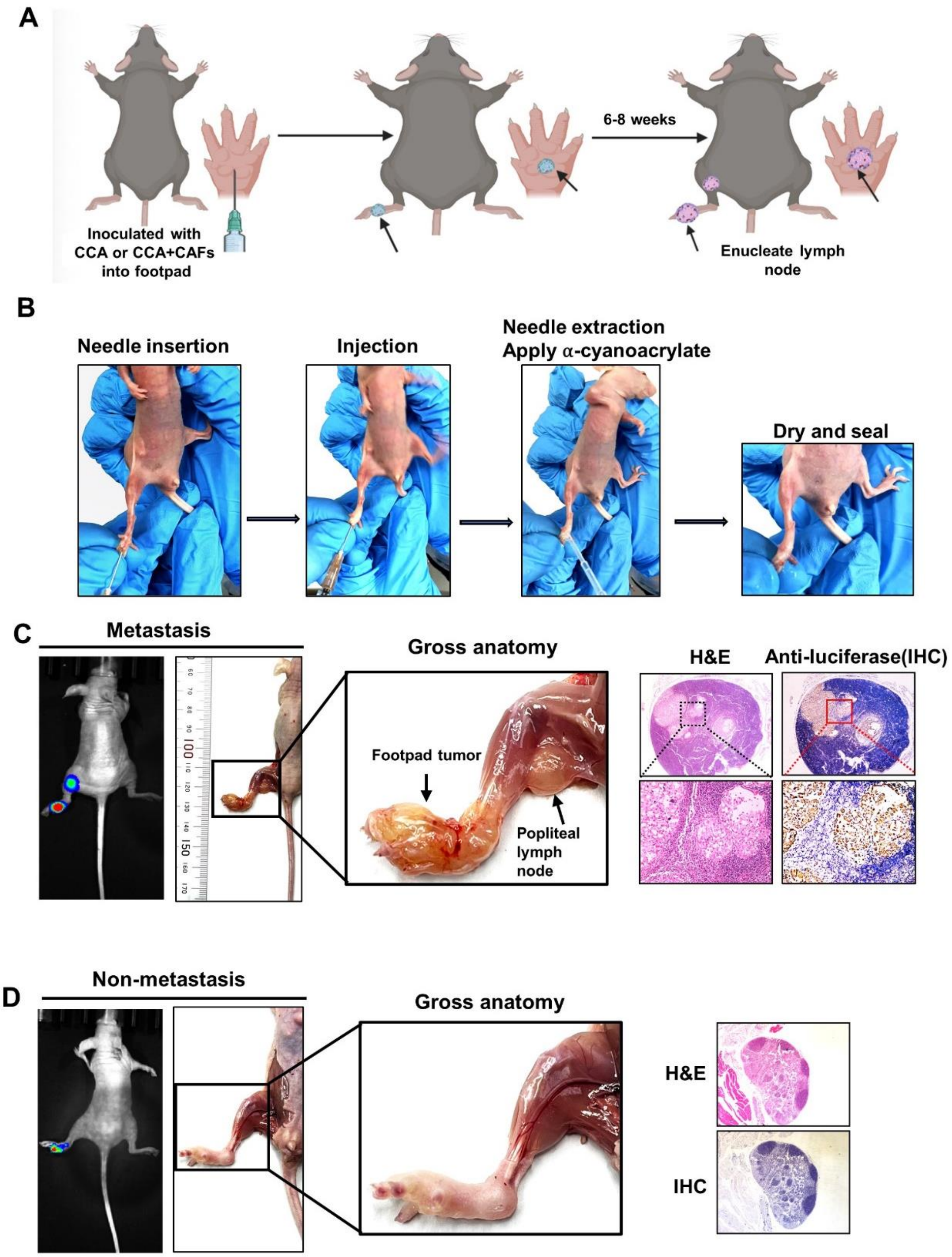

Supplementary Figure 12. Establishment and evaluation of PLM model in nude mice. (A) Schematic representation of the establishment of the nude mouse model of PLM. **(B)** The details of footpad injection of CCA cells. **(C)** Evaluation of positive PLNs. **(D)** Evaluation of negative PLNs.

© 2023. Yan J et al. Published online at http://www.aginganddisease.org/EN/10.14336/AD.2023.0420

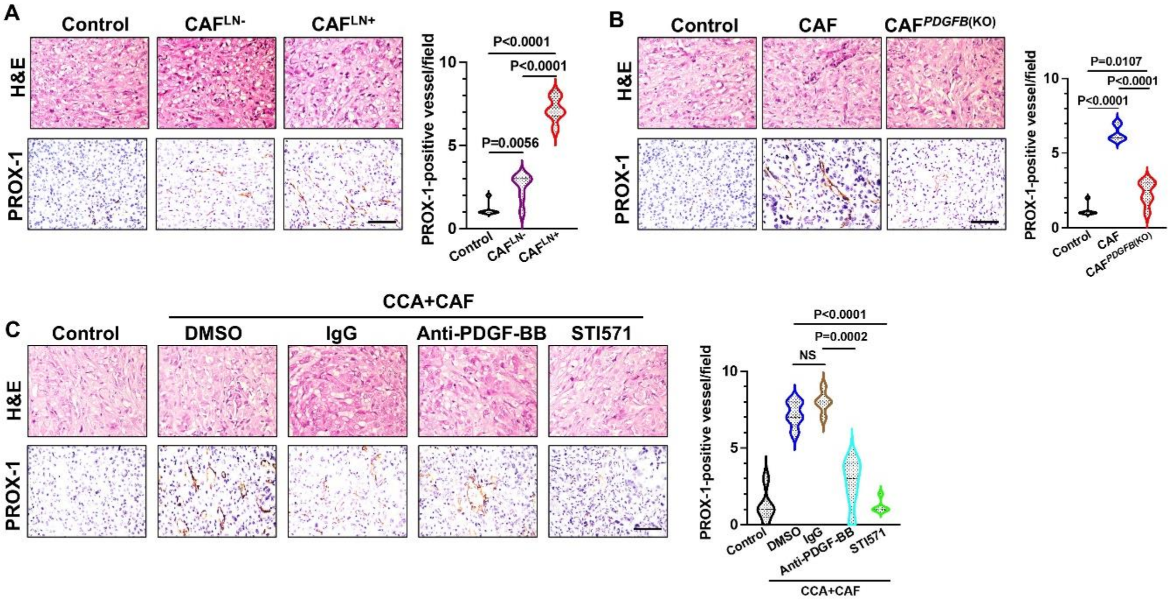

Supplementary Figure 13. CAFs promoted lymphangiogenesis *in vivo*. (A) Representative images of intratumoral microlymphatic vessels stained with anti-PROX-1 antibodies in the LN^{+}CAF and LN^{-}CAF groups. (B) Representative images of intratumoral microlymphatic vessels stained with anti-PROX-1 antibodies in the CAF and CAF$^{PDGFB(KO)}$ groups. (C) Representative images of intratumoral microlymphatic vessels stained with anti-PROX-1 antibodies in the DMSO, IgG, Anti-PDGF-BB antibodies, and STI571 groups. All *in vitro* experiments were performed with three biological replicates. The error bars indicate the SD of three independent experiments. Statistical method: One-way ANOVA. Scale bars: 200 μm.

© 2023. Yan J et al. Published online at http://www.aginganddisease.org/EN/10.14336/AD.2023.0420

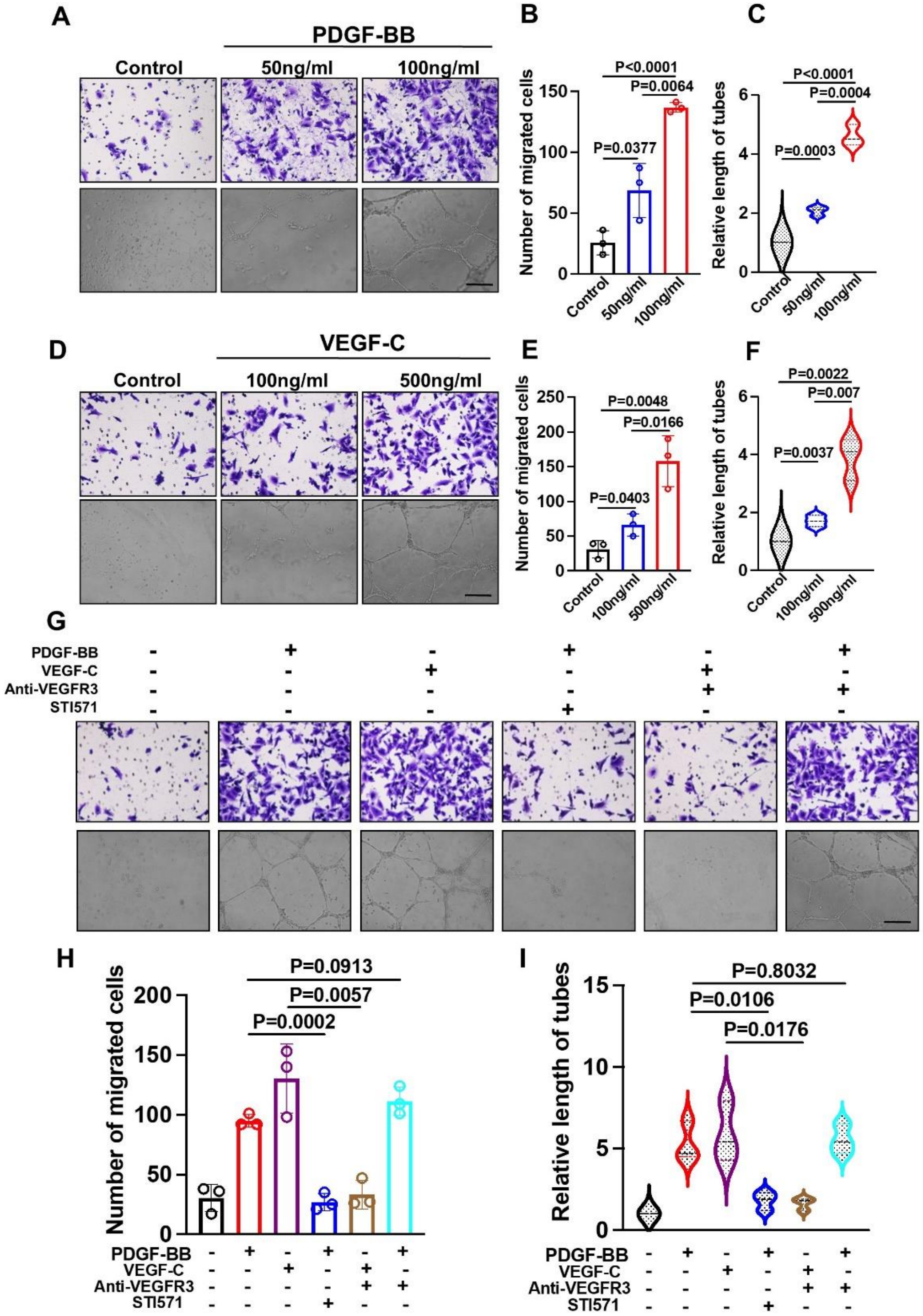

Supplementary Figure 14. PDGF-BB-induced tube formation and migration of HLECs independent of the VEGF-C/VEGFR-3 pathway *in vitro*. (A-C) Representative images **(A)** and quantification of Transwell migration **(B)** and tube formation **(C)** of HLECs treated with PDGF-BB at different concentrations. **(D-F)** Representative images **(D)** and quantification of Transwell migration **(E)** and tube formation **(F)** of HLECs treated with VEGF-C at different concentrations. **(G-I)** Representative images **(G)** and histogram analysis of Transwell migration **(H)** and tube formation **(I)** of HLECs treated with PDGF-BB (100 ng/ml), STI-571 (PDGFR-β inhibitor), VEGF-C (500 ng/ml), and Anti-VEGFR-3 antibodies. All *in vitro* experiments were performed with three independent experiments. The error bars indicate the SD of the mean. Statistical method: One-way ANOVA. Scale bars: 100 μm.

© 2023. Yan J et al. Published online at http://www.aginganddisease.org/EN/10.14336/AD.2023.0420

SUPPLEMENTARY DATA

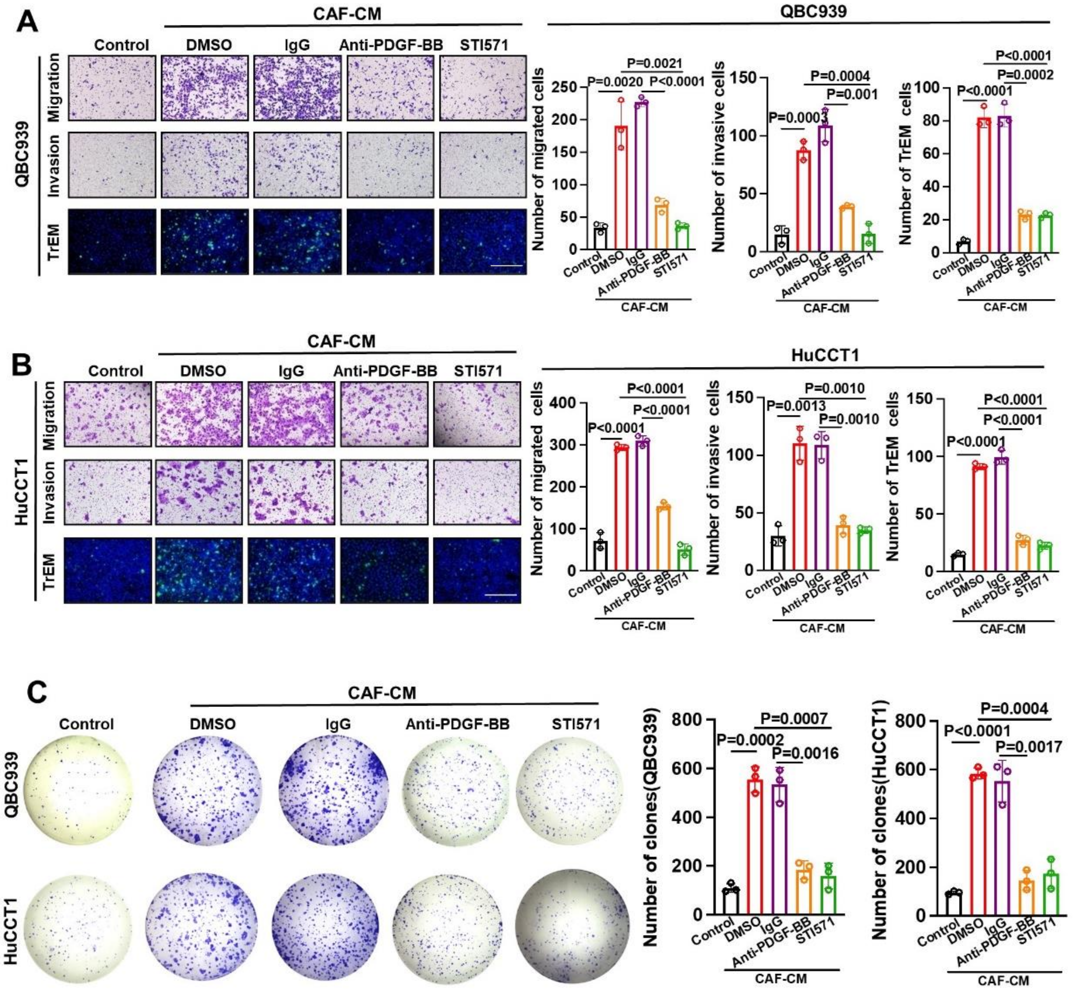

Supplementary Figure 15. CAF-derived PDGF-BB promoted CCA aggressiveness through PDGFR-β *in vitro*. (A, B) Representative images and histogram analysis of the migration, invasion, and TrEM assays for QBC939 **(A)** and HuCCT1 cells **(B)** incubated with CAF supernatant (CAF-CM) treated with DMSO, IgG, anti-PDGF-BB antibodies, or STI571. **(C)** Representative images and histogram analysis of QBC939 and HuCCT1 colony formation after incubation with CAF-CM treated with DMSO, IgG, anti-PDGF-BB antibodies, or STI571. All experiments were performed with three independent experiments. The error bars indicate the SD of the mean. Statistical method: One-way ANOVA. Scale bars: 100 μm.

© 2023. Yan J et al. Published online at http://www.aginganddisease.org/EN/10.14336/AD.2023.0420

SUPPLEMENTARY DATA

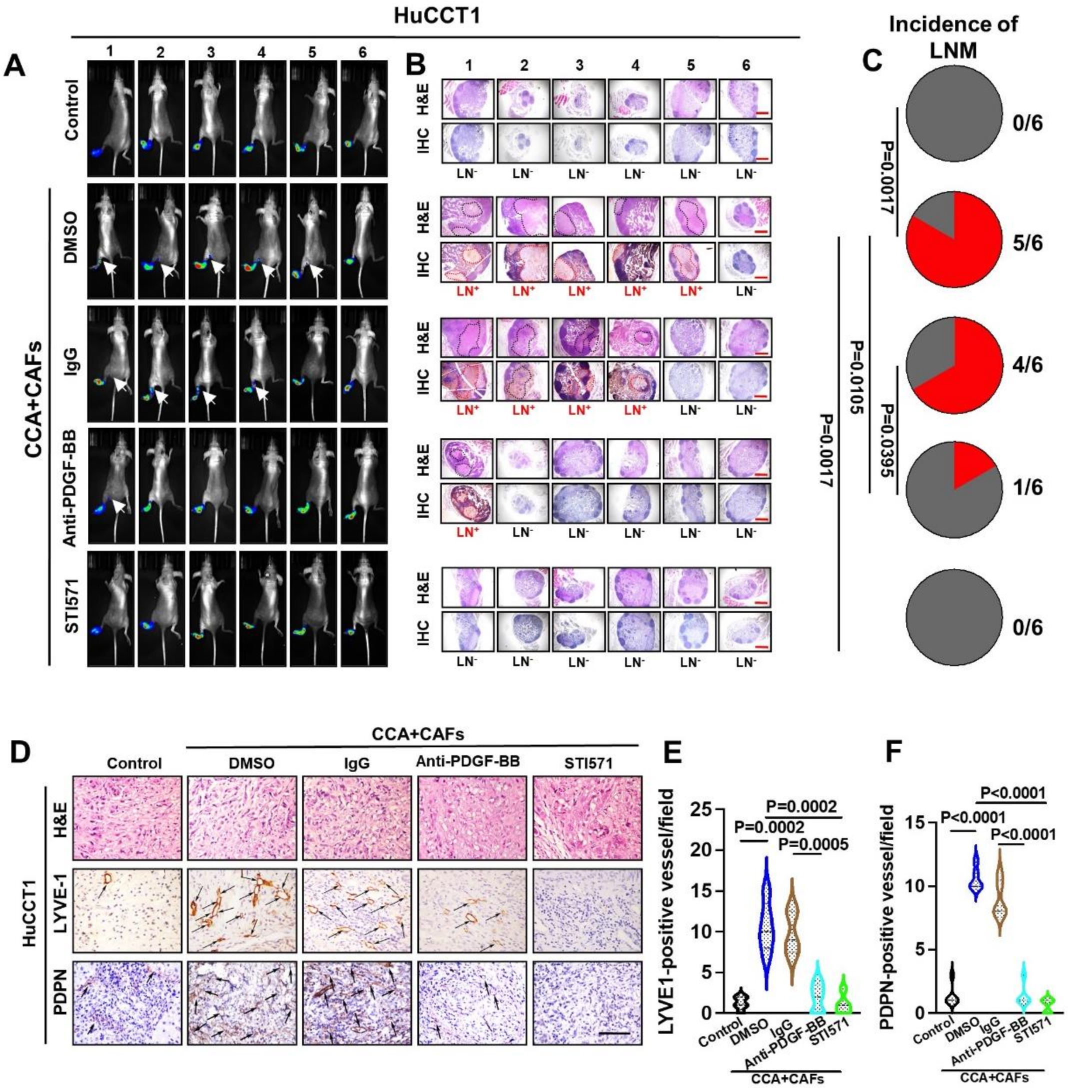

Supplementary Figure 16. CAFs promote lymphangiogenesis and LNM through the PDGF-BB/PDGFR-β pathway *in vivo*. (A) Representative images of bioluminescence analysis of footpad tumors and PLNM in HuCCT1 cells combined with CAFs on nude mice xenografts treated with DMSO, IgG, Anti-PDGF-BB, or STI571 (n = 6). **(B)** Enucleated PLNs and immunohistochemical staining of each PLN with anti-luciferase antibodies in the metastatic and non-metastatic groups. Scale bars: 50 μm. **(C)** Pie chart analysis of the PLNM rate of HuCCT1 cells. **(D-F)** Representative images of intratumoral microlymphatic vessels stained with anti-LYVE1 antibodies and PDPN (left, black arrows) **(D)** and histogram quantification of LMVD (right) in the HuCCT1 group **(E, F)**. Error bars represent the SD of the mean. All *in vitro* experiments were performed with three biological replicates or three independent experiments. The error bars indicate the SD three independent experiments. Statistical method: (E, F) One-way ANOVA, (C) χ^2 test. Scale bars: (B) 50 μm, (D) 200 μm.

© 2023. Yan J et al. Published online at http://www.aginganddisease.org/EN/10.14336/AD.2023.0420

SUPPLEMENTARY DATA

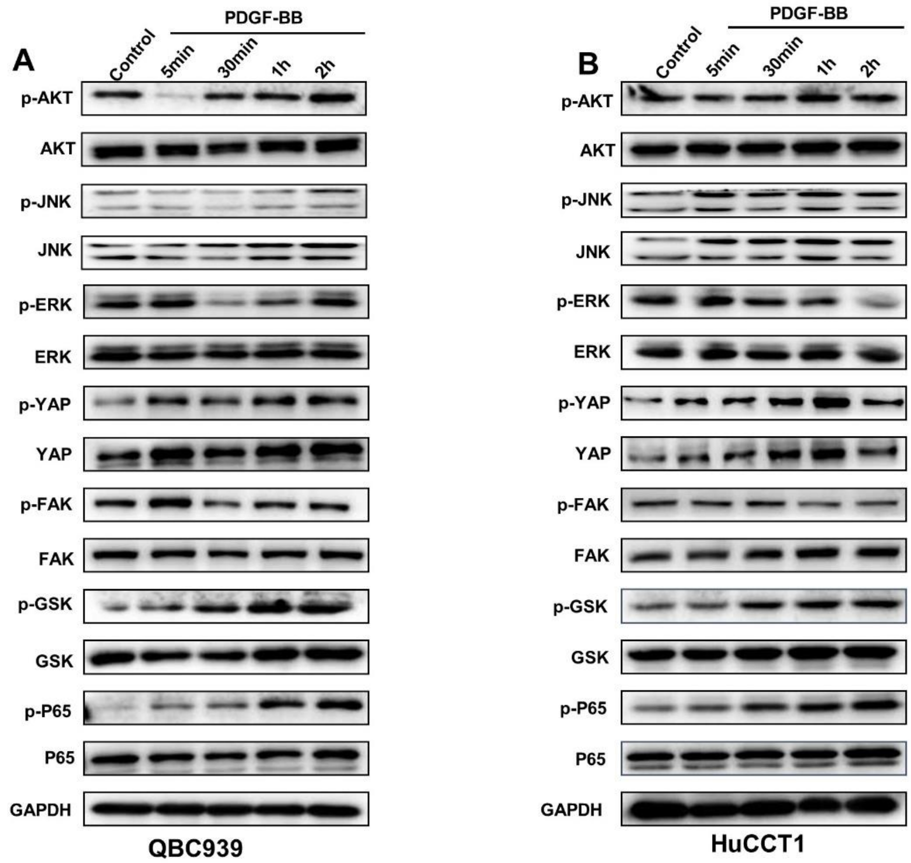

Supplementary Figure 17. PDGF-BB induces intracellular pathways in CCA cells *in vitro*. (A, B) Total and phosphorylated AKT, ERK, YAP, FAK, GSK, and P65 levels in both QBC939 **(A)** and HuCCT1 **(B)** cells, as detected by western blotting after PDGF-BB treatment.

© 2023. Yan J et al. Published online at http://www.aginganddisease.org/EN/10.14336/AD.2023.0420

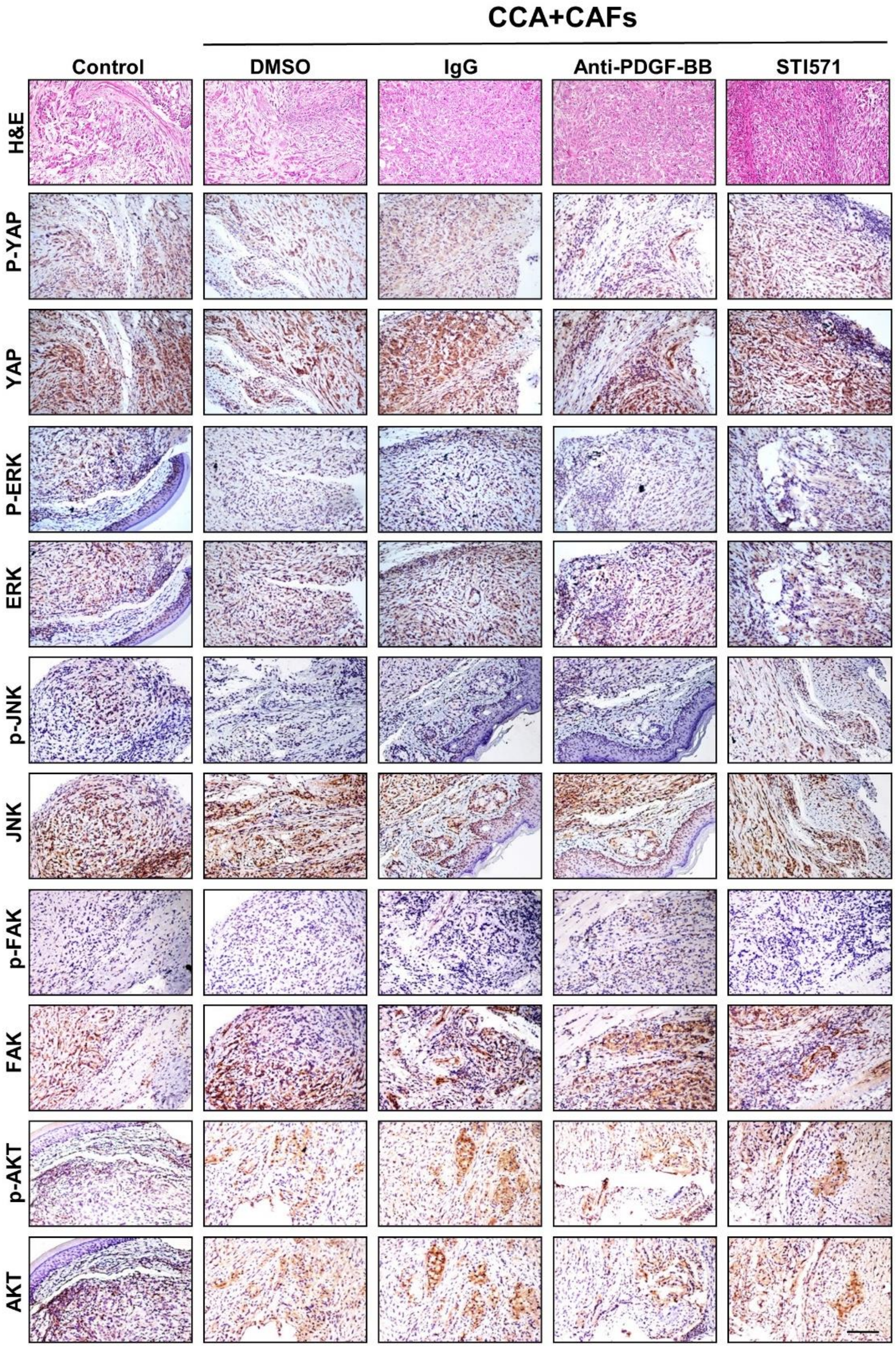

Supplementary Figure 18. CAF-derived PDGF-BB induces intracellular pathways in CCA cells *in vivo*. IHC staining showing that the FAK, p-FAK, YAP, p-YAP, JNK, p-JNK, ERK, p-ERK, AKT, and p-AKT levels in CCA cells were not activated in the indicated tumors. Scale bar: 100 µm.

© 2023. Yan J et al. Published online at http://www.aginganddisease.org/EN/10.14336/AD.2023.0420

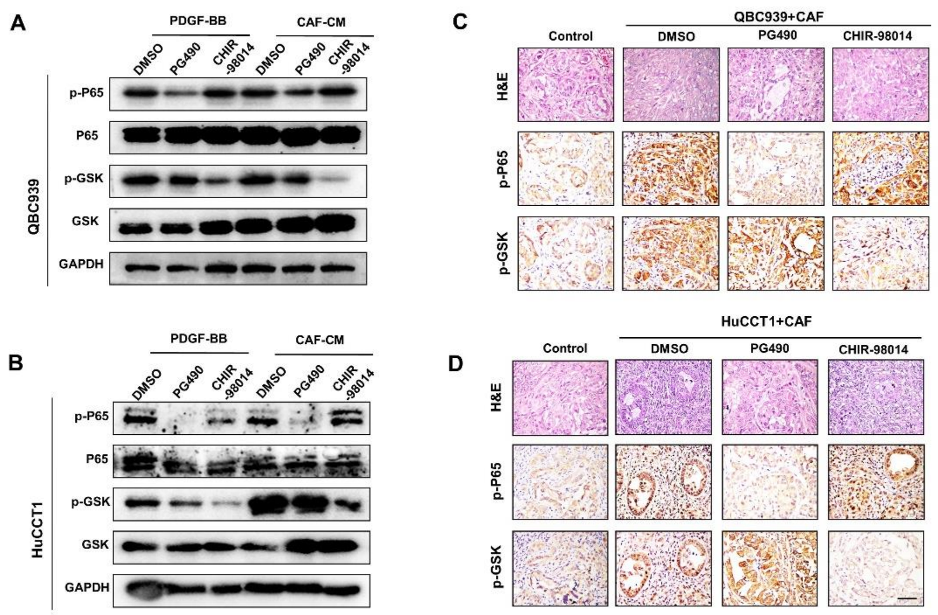

Supplementary Figure 19. GSK and P65 signaling pathways were inhibited by the corresponding pathway inhibitors *in vivo* and *in vivo*. (A, B) Representative western blotting results of GSK and P65 signaling pathway proteins in QBC939 **(A)** and HuCCT1 **(B)** cell lines treated with PDGF-BB and CAF supernatant treated with the corresponding pathway inhibitors. **(C, D)** Immunohistochemical staining of p-GSK and p-P65 in QBC939 **(C)** and HuCCT1 **(D)** tumors. n = 5 animals per group. Scale bar: 200 μm.

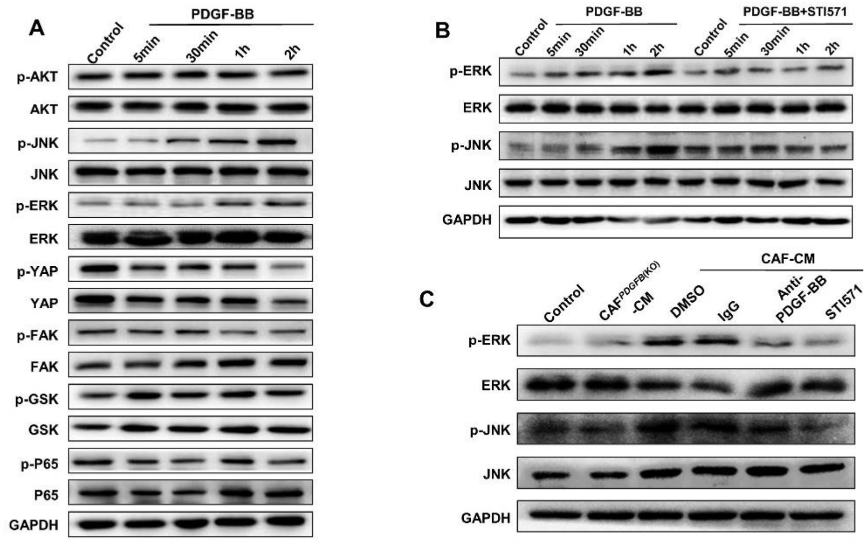

Supplementary Figure 20. PDGF-BB induces intracellular pathways in HLECs *in vitro*. (A) Representative western blotting results of total and phosphorylated AKT, JNK, ERK, YAP, FAK, GSK, and P65 levels in HLECs treated with PDGF-BB. **(B)** Representative western blotting results of total and phosphorylated JNK and ERK levels in PDGF-BB or PDGF-BB plus STI571-treated HLECs. **(C)** Representative western blotting results of total and phosphorylated JNK and ERK levels in HLECs treated with CAF$^{PDGFB\text{(KO)}}$ supernatant or CAF supernatant treated with DMSO, IgG, anti-PDGF-BB, or STI571.

© 2023. Yan J et al. Published online at http://www.aginganddisease.org/EN/10.14336/AD.2023.0420

SUPPLEMENTARY DATA

Supplementary Table 1. Clinicopathological data of 126 patients with CCA.

		Case number	Percentage (%)
**Age**			
	≤ 50	26	20.6
	> 50	100	79.4
**Sex**			
	Male	71	56.3
	Female	55	43.7
**Tumor size**			
	≤ 5 cm	61	48.4
	> 5 cm	65	51.6
**Differentiation degree**			
	Low	54	42.9
	Medium-high	72	57.1
**Lymph node**			
	Negative	65	51.6
	Positive	61	48.4
**Capsular invasion**			
	No	31	24.6
	Yes	95	75.4
**Nerve infiltration**			
	No	82	65.1
	Yes	44	34.9
**Cancer embolus**			
	No	89	70.6
	Yes	37	29.4

Supplementary Table 2. Details of antibodies used in this study.

Antibodies	Company	Host	Specificity	Product Number	Dilution
AKT	Cell signaling technology	Mouse	H, M, R, Mk	2920	1:1000 (WB), 1:300 (IHC)
p-AKT	Cell signaling technology	Rabbit	H, M, R, Mk	13038	1:1000 (WB), 1:300 (IHC)
FAK	Cell signaling technology	Rabbit	H, M, R, Mk, B, Pg	3285	1:1000 (WB), 1:500 (IHC)
p-FAK	Cell signaling technology	Rabbit	H,M,R,Hm,Pg	3283	1:1000 (WB),
p-FAK	Signalway Antibody	Rabbit	H, M, R	C90918Bio	1:200 (IHC)
P65	Cell signaling technology	Rabbit	H, M, R, Hm, Mk, Dg	8242	1:1000 (WB), 1:400 (IHC)
p-P65	Cell signaling technology	Rabbit	H, M, R, Hm, Mk, Pg	3033	1:1000 (WB)
p-P65	Abcam	Rabbit	H	Ab86299	1:300 (IHC)
JNK	Signalway Antibody	Rabbit	H, M, R	48335	1:1000 (WB)
JNK	Abcam	Rabbit	H, M, R	Ab76125	1:300 (IHC)
p-JNK	Cell signaling technology	Rabbit	H, M, R, Dm, Sc	4668	1:1000 (WB), 1:100 (IHC)
ERK	Cell signaling technology	Mouse	H, M, R, Mk, Mi, Z, B, Pg	4696	1:1000 (WB), 1:300 (IHC)
p-ERK	Cell signaling technology	Rabbit	H, M, R, Hm, Mk, Mi, Dm, Z, B, Dg, Pg, Sc	4370	1:1000 (WB), 1:300 (IHC)
YAP	Cell signaling technology	Rabbit	H, M, R, Hm, Mk	14074	1:1000 (WB), 1:300 (IHC)
p-YAP	Cell signaling	Rabbit	H, M, R	13008	1:1000 (WB), 1:300 (IHC)

© 2023. Yan J et al. Published online at http://www.aginganddisease.org/EN/10.14336/AD.2023.0420

	technology				
GSK	Cell signaling technology	Rabbit	H, M, R, Mk	12456	1:1000 (WB), 1:300 (IHC)
p-GSK	Cell signaling technology	Rabbit	H, M, R, Mk	9323	1:1000 (WB), 1:50 (IHC)
GAPDH	Cell signaling technology	Rabbit	H, M, R, Mk, B, Pg	2118	1:1000 (WB)
β-tubulin	Cell signaling technology	Rabbit	H, M, R, Mk, Z, B	2128	1:1000 (WB)
α-SMA	Servicebio	Mouse	H, M, R, Rab	GB13044	1:1000 (WB), 1:300 (IHC), 1:100 (IF)
α-SMA	Abcam	Mouse	H, M, R	Ab7817	1:20 (FC)
Podoplanin	Novus	Rabbit	H, M	NBP1-90211	1:1000 (WB), 1:200 (IHC), 1:100 (IF)
LYVE-1	Abcam	Rabbit	H, M	Ab281587	1:5000 (WB), 1:5000 (IHC), 1:2000 (IF)
PROX-1	Abcam	Rabbit	H, M, R	Ab199359	M：1:300 (IHC), 1:200 (IF)
PROX-1	Cell signaling technology	Rabbit	H	14963	H：1:1000 (IHC), 1:500 (IF)
PDGFR-β	Signalway Antibody	Rabbit	H, M, R	41327	1:1000 (WB), 1:300 (IHC), 1:200 (IF)
PDGFR-α	Signalway Antibody	Rabbit	H, M, R	33470	1:1000 (WB), 1:300 (IHC)
PDGF-BB	ThermoFisher	Rabbit	H, M, R	PA588272	1:1000 (WB), 1:200 (IHC), 1:50 (IF)
Vimentin	Cell signaling technology	Rabbit	H, M, R, Mk	5741	1:1000 (WB), 1:200 (IF)
FAP	Cell signaling technology	Rabbit	H	66562	1:1000 (WB), 1:200 (IF)
FAP	ThermoFisher	Mouse	H	BMS168	1:100 (FC)
CK-19	Abcam	Mouse	H	Ab20210	1:200 (IF)
CK-19	Abcam	Rabbit	H	Ab76539	1:20 (FC)
Fibronectin	Cell signaling technology	Rabbit	H	26836	1:200 (IF)
CD31	Abcam	Mouse	H	Ab9498	1:100 (IF)
CD31	Biolegend	Mouse	H	303118	1:100 (FC)
CD45	Abcam	Rabbit	H	Ab40763	1:100 (IF), 1:20 (FC)
Ki67	Servicebio	Rabbit	H, M, R	GB111499	1:1000 (IHC)
Luciferase	Merck millipore	Rabbit	All	AB3256-I	1:300 (IHC)
PDGF-BB	Gene Tex	Goat	H	GTX10845	Neutralizing/Inhibition
IgG	Gene Tex	Goat	H	GTX35039	Isotype contorl
Anti-Rabbit IgG, HRP-linked Antibody	Beyotime	Goat	Rab	A0208	1:50 (IHC)
Anti-mouse IgG, HRP-linked Antibody	Beyotime	Goat	M	A0192	1:50 (IHC)
Anti-Rabbit IgG H&L (HRP)	Abcam	Goat	Rab	Ab6721-1mg	1:5000 (WB)
Anti-Mouse IgG H&L (HRP)	Abcam	Goat	M	Ab6789-1mg	1:5000 (WB)
Cy3 conjugated Goat Anti-mouse IgG (H+L)	Servicebio	Goat	M	GB21301	1:500 (IF)
Alexa Fluor® 488-conjugated Goat Anti-Rabbit IgG (H+L)	Servicebio	Goat	Rab	GB25303	1:500 (IF)
TSA-AF488	Invitrogen	-	-	B40953	1:200 (TSA)
TSA-AF555	Invitrogen	-	-	B40955	1:200 (TSA)

Abbreviations: WB, Western blot; IHC, Immunohistochemistry; IF, Immunofluorescence; FC, Flow cytometry; TSA, Tyramide Signal Amplification. H, Human; M, Mouse; R, Rat; Hm, Hamster; Mk, Monkey; Vir, Virus; Mi, Mink; Dm, Drosophila melanogaster; Z, zebrafish; B, Bovine; Dg, Dog; Pg, Pig; Sc, Saccharomyces cerevisiae; Rab, Rabbit; All, All species.

SUPPLEMENTARY DATA

Supplementary Table 3. Univariate and multivariate analysis of OS in patients with CCA patients (n = 126).

	OS in the CCA cohort					
	Univariate			Multivariate		
	HR	95% CI	*P*-value	HR	95% CI	*P*-value
Age (> 50)	0.996	0.667–1.489	0.986			
Sex (Male)	0.868	0.580–1.297	0.489			
Tumor size (> 5 cm)	2.322	1.524–3.539	< 0.0001*	2.603	1.446–4.685	0.001*
Differentiation degree (Low)	0.652	0.435–0.977	0.038*	1.020	0.658–1.583	0.928
Lymph node (Positive)	4.59	2.998–7.029	< 0.0001*	7.195	1.178–43.95	< 0.0001*
Capsular invasion (Yes)	1.749	1.067–2.867	0.026*	1.02	0.592–1.759	0.942
Nerve infiltration (Yes)	2.408	1.588–3.653	< 0.0001*	2.54	1.612–4.003	< 0.0001*
Cancer embolus (Yes)	2.321	1.507–3.573	< 0.0001*	1.415	0.882–2.271	0.151
PDGFR-β (Epithelial positive)	2.334	1.539–3.539	< 0.0001*	0.870	0.500–1.514	0.662
PDGFR-β (Stroma medium/high expression)	4.168	2.732–6.358	< 0.0001*	1.724	1.123–4.262	< 0.0001*

© 2023. Yan J et al. Published online at http://www.aginganddisease.org/EN/10.14336/AD.2023.0420
